# Identification of a 5-gene signature panel for the prediction of prostate cancer progression

**DOI:** 10.1038/s41416-024-02854-w

**Published:** 2024-10-14

**Authors:** Michelle Shen, Fernando García-Marqués, Arvind Muruganantham, Shiqin Liu, James Robert White, Abel Bermudez, Meghan A. Rice, Kelsey Thompson, Chun-Liang Chen, Chia-Nung Hung, Zhao Zhang, Tim H. Huang, Michael A. Liss, Kenneth J. Pienta, Sharon J. Pitteri, Tanya Stoyanova

**Affiliations:** 1 Department of Molecular and Medical Pharmacology, University of California Los Angeles, Los Angeles, CA USA; 2 Department of Radiology, Stanford University, Stanford, CA USA; 3 Resphera Biosciences LLC, Baltimore, MD USA; 4 Department of Molecular Medicine, UT Health San Antonio, San Antonio, TX USA; 5 School of Nursing, UT Health San Antonio, San Antonio, TX USA; 6 Department of Urology, UT Health San Antonio, San Antonio, TX USA; 7 Brady Urological Institute, Johns Hopkins School of Medicine, Baltimore, MD USA; 8 Department of Urology, University of California Los Angeles, Los Angeles, CA USA

**Keywords:** Prognostic markers, Urological cancer

## Abstract

**Background:**

Despite nearly 100% 5-year survival for localised prostate cancer, the survival rate for metastatic prostate cancer significantly declines to 32%. Thus, it is crucial to identify molecular indicators that reflect the progression from localised disease to metastatic prostate cancer.

**Methods:**

To search for molecular indicators associated with prostate cancer metastasis, we performed proteomic analysis of rapid autopsy tissue samples from metastatic prostate cancer (*N* = 8) and localised prostate cancer (*N* = 2). Then, we utilised multiple independent, publicly available prostate cancer patient datasets to select candidates that also correlate with worse prostate cancer clinical prognosis.

**Results:**

We identified 154 proteins with increased expressions in metastases relative to localised prostate cancer through proteomic analysis. From the subset of these candidates that correlate with prostate cancer recurrence (*N* = 28) and shorter disease-free survival (*N* = 37), we identified a 5-gene signature panel with improved performance in predicting worse clinical prognosis relative to individual candidates.

**Conclusions:**

Our study presents a new 5-gene signature panel that is associated with worse clinical prognosis and is elevated in prostate cancer metastasis on both protein and mRNA levels. Our 5-gene signature panel represents a potential modality for the prediction of prostate cancer progression towards the onset of metastasis.

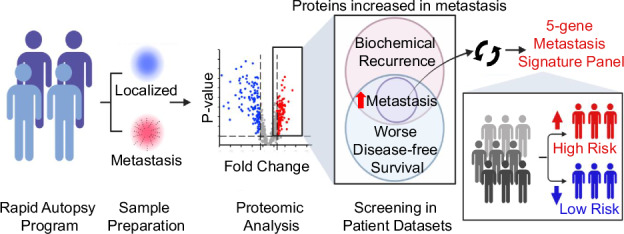

## Introduction

In 2024, there will be an estimated 299,010 new prostate cancer cases in the United States, making it the most common cancer among men [1]. Prostate cancer alone accounts for 29% of cancer incident cases in men, and 1 in 8 men are predicted to develop prostate cancer during their lifetime [1]. While 5-year relative survival for localised prostate cancer is as high as >99%, the 5-year relative survival for patients with metastatic prostate cancer is only 32% despite extensive research and new therapies [1, 2]. This suggests that metastatic prostate cancer accounts for approximately 35,250 deaths in the United States in 2024 alone, making prostate cancer one of the three leading causes of cancer-associated deaths amongst men despite the favourable prognosis for localised disease [1]. Statistical analysis also demonstrates a shift towards higher grade, higher stage prostate cancer, and an increased incidence of metastasis, most likely due to changes in screening guidelines [1, 3]. This increased prevalence of metastatic prostate cancer and the worse prognosis of these cases highlights the significant need to identify new predictors, drivers, and treatment strategies for these cancers.

Currently, radical prostatectomy, radiation therapy, active surveillance, and androgen deprivation therapy are the first line of treatment for localised prostate cancer [4, 5]. However, for patients with metastatic prostate cancer, the standard of care can also include second-generation anti-androgens, radiation therapy, and chemotherapies [4, 5]. The role of genetic alterations during the onset and progression of prostate cancer has been suggested in many studies [6–9]. Tests such as PCA3, SelectMDx, Decipher, and ConfirmMDx can also be used consecutively to assess the risk of prostate cancer [10–12]. However, with an increased incidence of prostate cancer metastasis-driven mortality, it is important to identify new markers that reflect metastasis progression, which can effectively identify patients who are at risk of faster progression and worse outcome. Therefore, the goal of this study is to identify new candidates that are associated with prostate cancer metastasis and disease outcome on both protein and mRNA levels so patients who most need escalated care can be identified expeditiously.

To identify candidates associated with prostate cancer metastasis, we performed proteomic analysis on rapid autopsy samples from localised prostate cancer tumours and prostate cancer metastases. Then, we utilised publicly available patient datasets to find the subset of these candidates that also correlate with worse clinical prognosis, including biochemical recurrence, reduced disease-free survival, and metastasis onset on the mRNA level. We discovered a new 5-gene signature panel that correlates with worse clinical prognosis and is elevated in prostate cancer metastasis. With the discovery of new protein and mRNA candidates that are associated with worse clinical prognosis and metastasis in prostate cancer, new therapeutic targets and prognostic predictors may arise to benefit prostate cancer patients with an increased risk of metastasis to reach optimal therapy selection.

## Methods

### Rapid autopsy samples

**Fig. 1:**
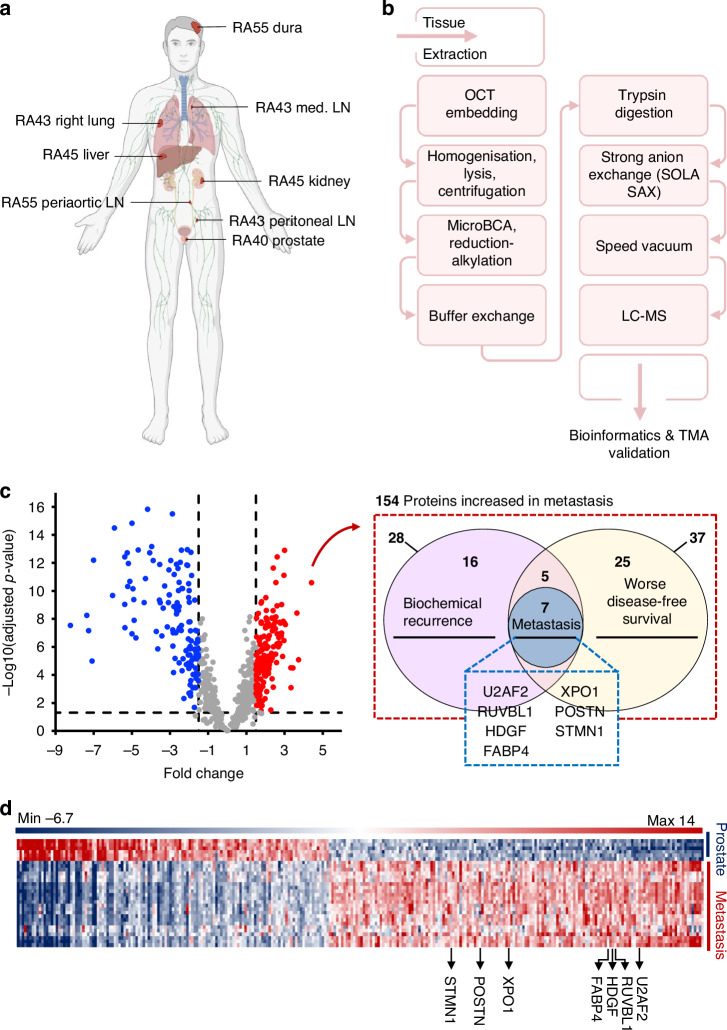
Proteomic analysis of rapid autopsy samples reveals candidates associated with prostate cancer metastasis. **a** Sketch represents the location from which the rapid autopsy samples were harvested. The patient ID and the corresponding location where the samples were collected are also labelled on the sketch. More information about the samples and the Rapid Autopsy Program can be found in Rubin et al., Mehra et al. and Drake et al. [14, 56, 57]. Created with BioRender.com (https://biorender.com). **b** The outline of the processing steps the rapid autopsy samples underwent, from tissue extraction, sample preparation for liquid chromatography–mass spectrometry (LC-MS) to the downstream bioinformatic analysis. **c** Volcano plot demonstrates the statistical significance (BH adjusted *P*-value) and fold change relative to localised prostate cancer. The FDR < 0.05 and fold change > |1.5| thresholds are plotted accordingly. Red represents proteins whose expression increased in the metastasis group relative to localised prostate cancer, and blue represents proteins whose expression decreased in the metastasis group relative to localised prostate cancer. The proteomic analysis revealed 154 candidates with increased expression in metastasis, and these candidates are analysed in publicly available datasets to characterise their association with worse clinical prognosis, including biochemical recurrence and disease-free survival. This discovered 12 candidates with positive correlations with the onset biochemical recurrence and worse patient disease-free survival outcome. Only the 7 proteins that also displayed elevated expression in prostate cancer metastasis relative to localised and normal prostate tissues were selected as candidates for the prostate cancer metastasis signature panel. **d** Heat map that shows the result of the proteomic analysis that compares localised prostate cancer tissues (*N* = 2) to tissues from prostate cancer metastasis (*N* = 8). Three injections are performed for each sample. Threshold of FDR < 0.05 and fold change > |1.5| are applied to reduce background noise of the analysis. The minimum and maximum levels of normalised expressions are labelled.

The rapid, “warm,” autopsy samples utilised in this study were collected from patients who died of androgen-independent, metastatic CRPC at the University of Michigan. Due to the short intervals between patient death and sample collection (average interval < 3 h), these tissue samples were characterised as “warm” or “rapid” autopsies. The sample collection was a part of the radical prostatectomy series under the Rapid Autopsy Program at the University of Michigan, which has been described previously [13, 14]. The protocol for the rapid autopsy programme was conducted with informed consent from the patient’s family or guardian and approved by the University of Michigan Institutional Review Board. This study utilised two samples of localised prostate tumours from patient R40 and eight prostate cancer metastasis samples from various sites of collection (Fig. 1a). One sample from the right lung, one sample from the peritoneal lymph node, and two samples from the mediastinal lymph node were collected from patient R43. One sample from the liver and one sample from the kidney were collected from patient R45. One sample from the periaortic lymph node and one sample from the dura were collected from patient R55 (Fig. 1a). Our sample size for localised prostate tumours is limited since localised prostate cancer samples are rare in rapid autopsy patients who died of metastatic CRPC. Clinical information for the four patients, including age at diagnosis, Gleason, and treatment exposure, are included in Table 1.

**Table 1:** Summary of the patient information.

Case no.	Age at diagnosis	Gleason score	Number of samples	Treatment^a^
RA40	72	10	2	H, C
RA43	47	9	4	P, R
RA45	66	N/A	2	N/A
RA55	73	9	2	H, C

Rubin et al. [14]; Mehra et al. [23].

*H* hormone ablation, *C* chemotherapy, *R* radiation, *P* radical prostatectomy.

^a^Treatment regimens.

### Sample preparation and proteomics

Optimal-Cutting-Temperature-Compound (OCT) was removed from the tissue samples by scraping, and samples were placed in pre-labelled 5 mL round bottom falcon tubes. Then, 1.0 mL of lysis buffer consisting of 12.5 mM Tris pH 8.0 (Fisher Scientific), 0.5 mM EDTA (EMD Inc.), 7.5 M urea (Sigma-Aldrich), and 1X protease inhibitor (Sigma-Aldrich) was added to the tissue samples and homogenised using a PRO-250 (ProScientific) Homogeniser probe on ice, followed by sonication using a Branson probe sonicator (Fisher Scientific). The insoluble fraction was pelleted by centrifuging tissue lysates at 14,000×*g* for 10 min at 4 °C. The supernatant was collected for protein quantification using a BCA protein assay kit (Thermo Scientific). An aliquot of 50 µg of protein from each tissue sample was processed for LC-MS-MS analysis. Tissue samples were prepared as follows: proteins were reduced with 2 µL of 200 mM Tris (2carboxyethyl) phosphine (TCEP) (Sigma-Aldrich) at a final concentration of 10 mM TCEP in solution, incubated at room temperature for 1 h, and vortexed occasionally. Then, free thiols on Cysteine residues were alkylated with iodoacetamide (Acros Organics) using a 1.5-fold molar excess of TCEP followed by incubation for 45 min at room temperature in the dark. Urea concentration was diluted to 300 mM using 50 mM ammonium bicarbonate (Sigma-Aldrich). Proteins were digested with sequencing-grade modified trypsin enzyme (Promega) in a 1:30 (enzyme: protein) ratio followed by incubation at 37 °C overnight. The resulting tryptic peptides were dried using a speed vacuum (LabConco) and desalted using Millipore ZipTip pipette tips (Millipore Sigma). Samples were dried and reconstituted in 50 µL of 0.1% formic acid (Fisher Scientific) in HPLC grade water (Fisher Scientific) for LC-MS analysis.

Two µg of tryptic peptides were loaded into a 20 µL sample loop and subsequently loaded onto an Acclaim PepMap C18 trap column (Thermo Fisher Scientific) in tandem using a Dionex Ultimate Rapid Separation Liquid Chromatography system (Thermo Fisher Scientific) at a rate of 5 µL/min for 10 min. Tryptic peptides were separated by reversed-phase chromatography on a 25 cm long C18 analytical column (New Objective) packed with Magic C18 AQ resin (Michrom Bioresources). Eluted peptides were ionised using a Nanospray flex ion source (Thermo Fisher Scientific) with 1.8 kV and introduced to an LTQ-Orbitrap Elite mass spectrometer (Thermo Fisher Scientific). The flow rate for the chromatography gradient was set at 0.6 µL/min with mobile phase A (consisting of 0.1% formic acid in water) set at 98% and mobile phase B (0.1% formic acid in acetonitrile) at 2% B for the first 10 min, slowly ramped up to 35% B over 100 min, followed by an increase to 85% B over 7 min with a 5-min hold. The analytical column was re-equilibrated before the next sample injection. Each sample was analysed in triplicate. The top 10 most abundant ions per MS1 scan were selected for higher energy collision-induced dissociation (35 eV) in a data-dependent fashion. MS1 resolution was set at 60,000, FT AGC target was set at 1e6, and the m/z scan range was set from m/z = 400–1800. MS2 AGC target at 3e4 and dynamic exclusion was enabled for 30 s.

### Proteomic statistical analysis

The resulting raw data files were searched using Byonic 2.11.0 (Protein Metrics) against the Swiss-prot reference human proteome databases (2017; 20,484 entries). The search setting included trypsin as the digestive enzyme, allowing up to two missed cleavages, and a precursor mass tolerance set at 10 parts per million (ppm). The search parameters also defined fixed modification of cysteine by carbamidomethylation and variable modifications for methionine oxidation and asparagine deamination. Peptide identifications were filtered with a 1% false discovery rate (FDR). Quantitative analysis was conducted on the MS1 level of all identified peptides using a custom R script, built upon the MSnbase package [15]. The relative protein quantities were initially computed relative to the average of the localised prostates group, followed by normalisation and standardisation. This process was performed using the Generic Integration Algorithm at the spectrum level, in line with the WSPP model [16]. Final statistical analysis was carried out using the Student’s *t*-test, and the adjusted *P*-values were computed using the Benjamini–Hochberg (BH) procedure. Only proteins with a *P*-value less than 0.05 and a fold change (FC) greater than |1.5| were considered for further analysis.

### Prostate cancer patient datasets for candidate screening

For the screening of proteomic-derived signature candidates, three independent and publicly available datasets were used. The mRNA expression z-scores of all 154 signature candidates whose proteomic expressions increased in metastases relative to localised prostate cancer were downloaded from the BS Taylor, *Cancer Cell* [17] and the TCGA, Firehose Legacy [18] datasets through cBioPortal (https://www.cbioportal.org/). The available sample-matched patient information, including biochemical recurrence status and patient disease-free survival, was also downloaded from the same datasets. After assessing the association with biochemical recurrence and disease-free survival outcome, 11 candidates were advanced to the next round of selection (Supplementary Fig. S1A, B). These 11 candidates advanced because they positively associated with prostate cancer biochemical recurrence and worse disease-free survival outcomes in at least one dataset with *P*-values of <0.01, or because their positive correlations (*P* < 0.05) with biochemical recurrence and worse disease-free survival are consistent in both datasets in either biochemical recurrence or worse disease-free survival (Supplementary Fig. S1B). The expression levels (in counts) of the 11 candidates were downloaded from the Chandran UR, *BMC Cancer*, [19] dataset (GDS2545, GSE6919 on Gene Expression Omnibus). The samples are then grouped based on the tissue of origin, including normal prostate tissues, benign prostate tissue that is adjacent to the tumour, localised prostate cancer tumour, and prostate cancer metastasis. The seven candidates that were highly expressed in metastasis relative to localised and normal prostate tissues were selected as the final candidates. Samples with missing expression data or clinical information were excluded from the analysis. Python code was utilised to systematically screen the 154 candidates, and selected candidates were inputted into the GraphPad Prism 10.0 software for plot generation. The Python code can be accessed via the GitHub repository (https://github.com/shen-michelle/5-gene-Metastasis.git).

### Kaplan–Meier survival curve

The Taylor BS et al., 2010, *Cancer Cell* [17] and the TCGA, Firehose Legacy [18] dataset were selected due to their large sample size (>100 samples per arm) and inclusivity of disease-free survival data. The mRNA z-scores of the signature candidates and the clinical information of patient disease-free outcome were obtained from previously published cBioPortal datasets (https://www.cbioportal.org/). Samples were grouped into high and low-expression groups using the median mRNA z-score expression as the cutoff. While screening the 154 proteomic-derived candidates, Kaplan–Meier curves were generated using the kaplanmeier-0.1.9 Python package. After screening, the Kaplan–Meier Survival Curves of the selected candidates were plotted using the GraphPad Prism 10.0 software, and the Log-rank *P*-value was computed to compare the disease-free survival outcome of the high and low-expression groups. To assess the association between the expression of the pooled signature panel and prostate cancer patient disease-free survival, a pooled expression value was computed assuming equal contributions of the normalised z-score expressions of all genes in the panel. For weighted models, the signature score was computed using coefficients for each of the 5 genes that are derived from elastic net model fitting. Then, the samples from the 2 datasets were separated into high and low-expression groups using the median cumulative expression score as the cutoff threshold. The Log-rank *P*-value, the hazard ratio (HR) with confidence interval, and the ${\chi }^{2}$ were all computed via GraphPad Prism 10.0.

### Principal component analysis (PCA)

Expression profiles (mRNA z-score) of the 7 candidates were downloaded from the TCGA Firehose Legacy dataset via cBioPortal (https://www.cbioportal.org/) [18]. Samples with missing expression information were excluded. Then, the data were compiled into a .csv file. The sklearn.preprocessing.StandardScaler Python package was used to standardise the data and the sklearn.decomposition.PCA Python package was used to perform the PCA analysis. The code is available in the GitHub repository (https://github.com/shen-michelle/5-gene-Metatasis-PCA.git).

### Assessment of the combined 5-gene signature panel in prostate cancer patient datasets

After screening, two additional analyses were performed to assess the expression profile of the 5-gene signature panel across various stages of prostate cancer. Grasso CS et al., *Nature*, 2012 (GSE35988) [20] and Varambally S et al., *Cancer Cell*, 2005 (GSE3325) [21] were accessed via Gene Expression Omnibus. The mRNA z-scores of the five genes that comprise the 5-gene signature panel were obtained from the two datasets, and the expression of the combined 5-gene panel was calculated assuming equal contributions from each gene. The relevant patient information was also downloaded from the datasets, and the samples were grouped based on the tissue of origin, including benign prostate, localised prostate cancer, and prostate cancer metastasis. Samples with missing expression data or clinical information were excluded from the analysis, and two-tailed Student’s *t*-tests were performed for the comparison of two groups, and plots display mean ± SD. In addition, the 5-gene signature panel was also assessed in the Gerhauser, *Cancer Cell*, 2018 dataset to test its association with biochemical recurrence [22]. Clinical information regarding biochemical recurrence and mRNA profiles were downloaded from cBioPortal (https://www.cbioportal.org/). This dataset contains 81 non-recurrent and 24 recurrent prostate cancer samples.

### Receiver-operating characteristic (ROC) and area under the curve (AUC)

Using the independent Grasso CS, *Nature*, 2012 dataset (GSE35988), the expression profiles (z-score) of the genes in the signature panel were downloaded via Gene Expression Omnibus. The combined 5-gene expression is calculated by averaging the expressions of the single candidates. The expressions of the single candidates and the combined 5-gene panel were used to generate ROC plots using the GraphPad Prism 10.0 software. Prostate cancer patients with metastasis (*N* = 39) are compared against prostate cancer patients with localised diseases (*N* = 59). Samples with missing expression data (*N* = 2 for RUVBL1, *N* = 41 for FABP4, and *N* = 4 for POSTN) were treated as zeros in the combined expression profile. The AUC and *P*-values for each plot were computed using the GraphPad Prism 10.0 software.

### Expression pattern across various Gleason scores

The mRNA expression z-scores of the candidates were obtained from the TCGA Firehose Legacy dataset via cBioPortal (https://www.cbioportal.org/) [18]. The sample-matched patient Gleason scores at radical prostatectomy were also obtained from the same dataset. The expression profiles of all candidates were then plotted across the various Gleason scores, and Gleason scores of 9 and 10 were grouped into Gleason score 9+ due to the limited sample size. Then, Student’s *t*-tests were performed to compare the expression of the candidate genes across each group of Gleason scores. Samples with missing Gleason score information or gene expression information were excluded from the analysis. The *P*-values were computed using GraphPad Prism 10.0.

### Elastic net model fitting for weighted signature score

The glmnet package in R was used to perform elastic net model fitting using the five signature genes (U2AF2, RUVBL1, HDGF, FABP4, and STMN1) as variables. Three elastic net models were generated. Model 1 was trained on the TCGA, Firehose Legacy dataset (with 400 non-recurrent and 91 recurrent prostate cancer samples). Model 2 was trained on the BS Taylor, *Cancer Cell*, 2010 dataset (with 104 non-recurrent and 36 recurrent prostate cancer samples). Model 3 was trained on the two datasets combined. The area under the curve (AUC) was computed, and the coefficients for each of the five signature genes were obtained from the final models. For Model 1, AUC = 65.5 and the coefficients are 0.21995 for U2AF2, 0.11618 for RUVBL1, −0.06077 for HDGF, 0 for FABP4, and 0.31602 for STMN1. For Model 2, AUC = 79.89 and the coefficients are −0.41372 for U2AF2, 0.15510 for RUVBL1, 0.20957 for HDGF, 0.31032 for FABP4, and 0.45953 for STMN1. For Model 3, the AUC = 67.64 and the coefficients are 0.04864 for U2AF2, 0.09297 for RUVBL1, −0.02813 for HDGF, 0.06348 for FABP4, and 0.41772 for STMN1. The code created for this model fitting can be accessed via the GitHub repository (https://github.com/shen-michelle/5-gene-Metastasis-Weights).

### Statistical analysis

Student’s *t*-tests were performed using the GraphPad Prism 10.0 software to compare the means of the two groups. Equal variance was assumed between comparison groups. The plots display the mean ± SD, and the corresponding *P*-values were labelled accordingly. The Log-rank (Mantel-Cox) test was performed for all Kaplan–Meier analyses using the GraphPad Prism 10.0 software. The Chi-square (${\chi }^{2}$) statistic and the *P*-value were calculated from the Log-rank test to assess the statistical significance of the outcome prediction. The hazard ratios (HR) with 95% confidence intervals were computed using the Mantel-Haenszel method to compare the risk of worse disease-free survival outcomes in the high-expression groups relative to the low-expression groups. For the receiver-operating characteristic (ROC) analyses, the GraphPad Prism 10.0 software was used to compute the curves and calculate the area under the curve (AUC). For all plots generated, ns = non-significant, *P < 0.05, **P < 0.01, ***P < 0.001, ****P < 0.0001.

## Results

### Proteomic profiling of rapid autopsy patient samples reveals candidates associated with prostate cancer metastasis

To identify a signature panel that characterises prostate cancer metastasis, we performed proteomic analysis on rapid autopsy samples (two localised prostate cancer and eight prostate cancer metastasis samples). These samples were obtained from four patients who were diagnosed with androgen-independent metastatic castration-resistant prostate cancer (mCRPC) [14, 23]. The clinical information of these four patients is described in Table 1 and includes age at diagnosis, Gleason score, and treatment history. The samples utilised in this study were part of the Rapid Autopsy Program at the University of Michigan. The eight samples of prostate cancer metastases were collected from various metastatic sites, including one sample from the right lung, one sample from the peritoneal lymph node, two samples from the mediastinal lymph node, one sample from liver, one sample from kidney, one sample from the periaortic lymph node, and one sample from the dura (Fig. 1a). Samples were subjected to flash freezing and prepared for liquid chromatography–mass spectrometry (LC-MS) (Fig. 1b). Each of the ten rapid autopsy samples were analysed in triplicate by LC-MS, and the protein expression profiles of the metastasis samples were compared to the protein profile of the localised prostate cancer group. The adjusted *P*-values were computed using the Benjamini–Hochberg (BH) procedure.

To reduce the background signal of the proteomic results and to increase the relevance of the proteomic analysis, a threshold false discovery rate (FDR) of *P* < 0.05 and a threshold fold change (FC) of FC > |1.5| were applied to the proteomic results, revealing 154 protein candidates with increased levels in the metastasis group relative to the localised prostate group, and 129 candidates with decreased levels in the metastasis group (Fig. 1c, d). To select metastasis candidates that capture the worse clinical prognosis of prostate cancer metastasis, we utilised multiple publicly available prostate cancer patient datasets to further screen the 154 proteins with increased expression in the metastasis group (Supplementary Fig. S1A). We assessed these 154 proteins in the TCGA Firehose Legacy dataset and the BS Taylor, *Cancer Cell*, [17, 18] dataset to search for candidates that correlate with prostate cancer biochemical recurrence and predict shorter time of disease-free survival (Supplementary Fig. S1A). Elevated levels of 28 candidates from the 154 proteins were identified to correlate with prostate cancer recurrence in at least one of the two datasets, and increased levels of 37 protein candidates correlate with worse disease-free survival in at least one of the two datasets (Fig. 1c). This identified 12 candidates that positively correlated with biochemical recurrence and shorter disease-free survival in at least one dataset (Fig. 1c). To reduce dataset-specific candidates, a selection criterion was set to include candidates that displayed at least one set of consistent positive correlations (*P* < 0.05) in both datasets (RUVBL1, HDGF, POSTN, STMN1, ASPN, CA2, H2AC1) (Supplementary Fig. S1B). Candidates that were implicated in a single dataset (U2AF2, FABP4, XPO1, DDX39B) were only included in further analyses if they satisfied a more stringent FDR of *P* < 0.01 in their association with biochemical recurrence and worse disease-free survival (Supplementary Fig. S1B).

Then, these 11 candidates were further analysed in the Chandran UR, *BMC Cancer*, [19] dataset to discover candidates associated with metastasis relative to localised prostate cancer and normal samples in these datasets [17, 19] (Supplementary Fig. S1A, B). This led to the identification of 7 genes that fit these criteria (U2AF2, RUVBL1, HDGF, FABP4, XPO1, POSTN, and STMN1) (Fig. 1c, d). These 7-gene candidates were chosen due to their elevated levels in metastatic prostate cancer in both protein and mRNA levels and their association with worse clinical prognosis in terms of increased risk of biochemical recurrence and worse patient disease-free survival outcome (Supplementary Fig. S1A, B).

### Elevated levels of the 7-gene candidates, U2AF2, RUVBL1, HDGF, FABP4, XPO1, POSTN, and STMN1, correlate with prostate cancer biochemical recurrence and worse patient disease-free survival

**Fig. 2:**
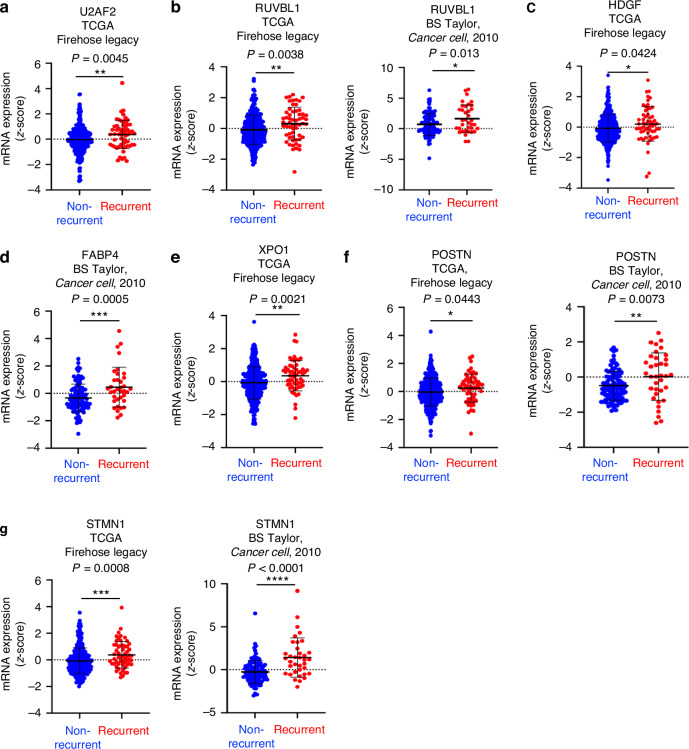
The 7 candidates, U2AF2, RUVBL1, HDGF, FABP4, XPO1, POSTN, and STMN1, are highly expressed in recurrent prostate cancer relative to non-recurrent prostate cancer. **a** Scatter dot plot shows the mRNA expression of U2AF2 in recurrent prostate cancer (*N* = 58) and non-recurrent prostate cancer (*N* = 371) from the TCGA Firehose Legacy dataset. **b** Scatter dot plots show the mRNA expression of RUVBL1 in recurrent vs non-recurrent prostate cancer from the TCGA Firehose Legacy dataset (left) and the BS Taylor, *Cancer Cell*, 2010 dataset (right). In the BS Taylor, *Cancer Cell*, 2010 dataset, *N* = 36 for recurrent prostate cancer and *N* = 104 for non-recurrent prostate cancer. **c** Scatter dot plot of HDGF mRNA expression profile in recurrent vs non-recurrent prostate cancer from the TCGA Firehose Legacy dataset. **d** Scatter dot plot of FABP4 mRNA expression profile in recurrent vs non-recurrent prostate cancer from the BS Taylor, *Cancer Cell*, 2010 dataset. **e** Scatter dot plot of XPO1 mRNA expression profile in recurrent vs non-recurrent prostate cancer from the TCGA Firehose Legacy dataset. **f** Scatter dot plots of POSTN mRNA expression levels in recurrent vs non-recurrent prostate cancer in the TCGA Firehose Legacy dataset (left) and the BS Taylor, *Cancer Cell*, 2010 dataset (right). **g** Scatter dot plots of STMN1 mRNA expressions in recurrent and non-recurrent prostate cancer in the TCGA Firehose Legacy dataset (left) and the BS Taylor, *Cancer Cell*, 2010 dataset (right). The Student’s *t*-test was performed with *P < 0.05, **P < 0.01, ***P < 0.001, and ****P < 0.0001 for all comparisons between the two groups. The *P*-values are labelled correspondingly on each of the scatter dot plots.

The 7-gene candidates we identified demonstrated a positive correlation with recurrent prostate cancer in at least one of the two datasets with clinical information of biochemical recurrence (Fig. 2). In the TCGA Firehose Legacy dataset, U2AF2 (*P* = 0.0045), RUVBL1 (*P* = 0.0038), HDGF (*P* = 0.042), XPO1 (*P* = 0.0021), POSTN (*P* = 0.044), and STMN1 (*P* = 0.0008) were elevated in the recurrent group (*N* = 58) relative to the non-recurrent group (*N* = 371) (Fig. 2a, b-left, c, e, f-left, g-left). In the BS Taylor, *Cancer Cell*, 2010 dataset, RUVBL1 (*P* = 0.013), FABP4 (*P* = 0.0005), POSTN (*P* = 0.0073), and STMN1 (*P* < 0.0001) were significantly elevated in recurrent prostate cancer (*N* = 36) relative to non-recurrent prostate cancer (*N* = 104) (Fig. 2b-right, d, f-right, g-right). There was no difference in FABP4 levels between the non-recurrent and recurrent groups in the TCGA Firehose Legacy dataset (Supplementary Fig. S2A). There was also no significant difference in U2AF2, HDGF, and XPO1 levels between the non-recurrent and recurrent groups in the BS Taylor, *Cancer Cell*, 2010 dataset, potentially due to a smaller sample size (Supplementary Fig. S2B–D).

**Fig. 3:**
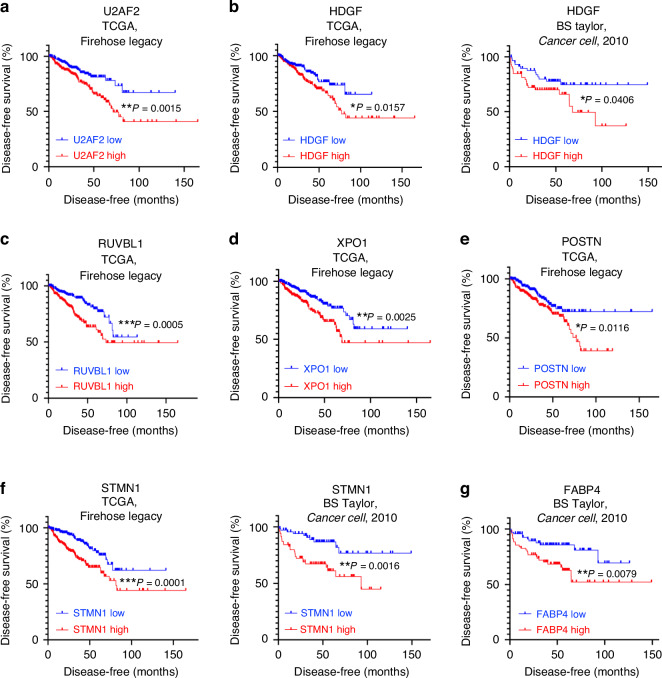
High expressions of the 7 candidates correspond with worse patient disease-free survival in prostate cancer patients. **a** Kaplan–Meier plot of prostate cancer disease-free survival outcomes based on high and low U2AF2 expressions in the TCGA Firehose Legacy dataset. The high (*N* = 246) and low (*N* = 245) groups were determined using the median expression level of U2AF2 as the cutoff threshold. **b** Kaplan–Meier plots of HDGF expressions and patient disease-free survival outcome in the TCGA Firehose Legacy dataset (left) and the BS Taylor, *Cancer Cell*, 2010 dataset (right). In the BS Taylor, *Cancer Cell*, 2010 dataset, *N* = 70 for both HDGF high and HDGF low groups. The groups were determined using the median HDGF expression level as cutoff. **c** Kaplan–Meier plot of RUVBL1 expression and prostate cancer patient disease-free survival outcome in the TCGA Firehose Legacy dataset. **d** Kaplan–Meier plot of XPO1 expression and prostate cancer patient disease-free survival outcome in the TCGA Firehose Legacy dataset. **e** Kaplan–Meier plot shows the correlation between POSTN expression and patient disease-free survival outcome in the TCGA Firehose Legacy dataset. **f** Kaplan–Meier plots show the correlation between STMN1 expression and prostate cancer patient disease-free survival outcome in both the TCGA Firehose Legacy dataset (left) and BS Taylor, *Cancer Cell*, 2010 dataset (right). **g** Kaplan–Meier plot of FABP4 expression and prostate cancer patient disease-free survival outcome in the BS Taylor, *Cancer Cell*, 2010 dataset. For all Kaplan–Meier plots, the Log-rank *P*-values are computed and labelled on the corresponding plots. *P < 0.05, **P < 0.01, and ***P < 0.001.

In addition, increased expression of these 7-gene candidates was also associated with worse prostate cancer disease-free survival in either the TCGA Firehose Legacy dataset and/or the BS Taylor, *Cancer Cell*, 2010 dataset (Fig. 3) [17, 18]. Patient disease-free survival was selected as an inclusion criteria since metastasis is the major contributor to prostate cancer-driven mortality. Our results identified that an increased level of U2AF2 was associated with worse disease-free survival in the TCGA, Firehose Legacy dataset with *P* = 0.0015 (Fig. 3a). The median expression of U2AF2 was used as the cutoff threshold to determine the U2AF2 high (*N* = 246) and U2AF2 low (*N* = 245) groups. HDGF, RUVBL1, XPO1, POSTN, and STMN1 also displayed the same trend with *P* = 0.0157, 0.0005, 0.0025, 0.0116, and 0.0001 respectively (Fig. 3b-left, c–e, f-left). In the BS Taylor, *Cancer Cell*, 2010 dataset, elevated levels of HDGF, STMN1, and FABP4 correlated with worse disease-free survival with *P* = 0.0429, 0.0016, and 0.0079 respectively (Fig. 3b-right, f-right, g). The median expression levels of each candidate were also used as the cutoff threshold, and 70 samples were included in each of the high and low-expression groups. Due to variations between datasets, FABP4 expression did not predict patient disease-free survival in the TCGA Firehose Legacy dataset, while U2AF2, RUVBL1, XPO1, and POSTN did not predict disease-free survival in the BS Taylor, *Cancer Cell*, 2010 dataset (Supplementary Fig. S2E–I). In addition, six of the seven candidates (all except FABP4) were also associated with higher Gleason scores when assessed in the TCGA Firehose Legacy dataset (Supplementary Fig. S3). Notably, U2AF2 and STMN1 could differentiate between all Gleason scores ranging from 6 to 9+ (Supplementary Fig. S3A, G). This further suggests an association between increased expression of the candidates and worse clinical risks and prognosis. The statistically significant correlation between these 7-gene candidates and clinical prognostic factors such as biochemical recurrence and patient disease-free survival suggests their clinical potential as prognosis indicators for worse outcomes.

### The 7-gene candidates are elevated in metastatic prostate cancer

**Fig. 4:**
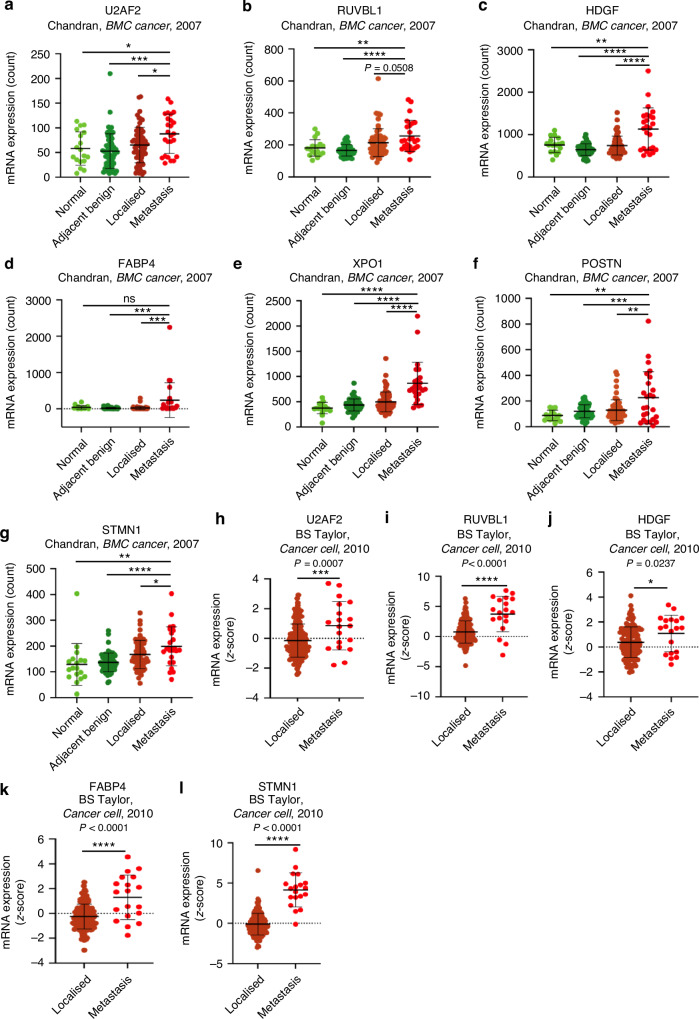
The 7 candidates are highly expressed in prostate cancer metastasis relative to localised prostate cancer and normal prostate tissues. **a** Expression profiles of U2AF2 in the Chandran, *BMC Cancer*, 2007 dataset (*N* = 18 for normal prostate tissues, *N* = 63 for normal prostate tissues adjacent to tumour, *N* = 65 for localised prostate cancer tumours, and *N* = 25 for metastatic prostate cancer). **b**–**g** Expression profiles of RUVBL1 (**b**), HDGF (**c**), FABP4 (**d**), XPO1 (**e**), POSTN (**f**), and STMN1 **g** in the Chandran, *BMC Cancer*, 2007 dataset described in (**a**). **h** Expression levels of U2AF2 in localised prostate cancer tumours vs prostate cancer metastases using the BS Taylor, *Cancer Cell*, 2010 dataset (*N* = 131 for localised prostate cancer samples and *N* = 19 for prostate cancer metastasis samples). **i**–**l** The expression levels of RUVBL1 (**i**), HDGF (**j**), FABP4 (**k**), and STMN1 (**l**) in localised prostate tumours vs prostate cancer metastases as described in (**h**). For all comparisons between the two groups, Student’s *t*-test was performed with ns = non-significant, *P < 0.05, **P < 0.01, ***P < 0.001, and ****P < 0.0001.

In addition, the levels of the 7-gene candidates were increased in metastasis samples relative to localised prostate cancer, benign prostate tissue adjacent to cancer, and normal prostate tissues (Fig. 4). The Chandran UR, *BMC Cancer*, 2007 dataset included 18 normal prostate tissues, 63 benign prostate tissues adjacent to tumour, 65 localised prostate cancer, and 25 prostate cancer metastasis [19]. In this dataset, all the 7-gene candidates exhibited a significant increase of mRNA expression in the metastasis group relative to localised tumours, with RUVBL1 exhibiting near statistical significance (U2AF2 *P* = 0.0106, RUVBL1 *P* = 0.051, HDGF *P* < 0.0001, FABP4 *P* = 0.0005, and STMN1 *P* = 0.032) (Fig. 4a–g). In addition to differentiating between metastasis and localised groups, the candidates also demonstrated the ability to stratify between normal and metastatic groups and between begin adjacent to tumour tissues and metastatic groups. The expressions of U2AF2 (*P* = 0.014, *P* = 0.0001), RUVBL1 (*P* = 0.005, *P* < 0.0001), HDGF (*P* = 0.0038, *P* < 0.0001), XPO1 (*P* < 0.0001, *P* < 0.0001), POSTN (*P* = 0.0068, *P* = 0.0002), and STMN1 (*P* = 0.0058, *P* < 0.0001) were all significantly elevated in the metastasis group relative to both normal prostate and benign adjacent to tumour groups (Fig. 4a–g). However, while the increased expression of FABP4 was statistically significant between metastasis and benign tissues adjacent to the tumour (*P* = 0.0004), this difference was not statistically significant between the metastasis and the normal group (*P* = 0.088) (Fig. 4d).

To further assess the positive association between the 7-gene candidates and prostate cancer metastasis, we also compared their expressions in the BS Taylor, *Cancer Cell*, 2010 (*N* = 131 for localised prostate cancer samples and *N* = 19 for metastasis samples). In the analysis of this dataset, five of the seven candidates displayed increased expression in the metastasis group relative to localised prostate cancer with U2AF2 *P* = 0.0007, RUVBL1 *P* < 0.0001, HDGF *P* = 0.0237, FABP4 *P* < 0.0001, and STMN1 *P* < 0.0001 (Fig. 4h–l) [17]. The difference in expression levels of XPO1 and POSTN did not reach statistical significance, likely due to the relatively small sample size of the metastasis group (*N* = 19) (Supplementary Fig. S2J–K). These results indicate that the 7-gene candidates selected have the potential to distinguish metastatic prostate cancer from localised prostate cancer. This, coupled with their association with worse clinical prognosis, suggests their clinical potential to assist in prognosis prediction and therapy selection.

### Novel 5-gene signature panel predicts worse patient disease-free survival relative to individual candidates

**Fig. 5:**
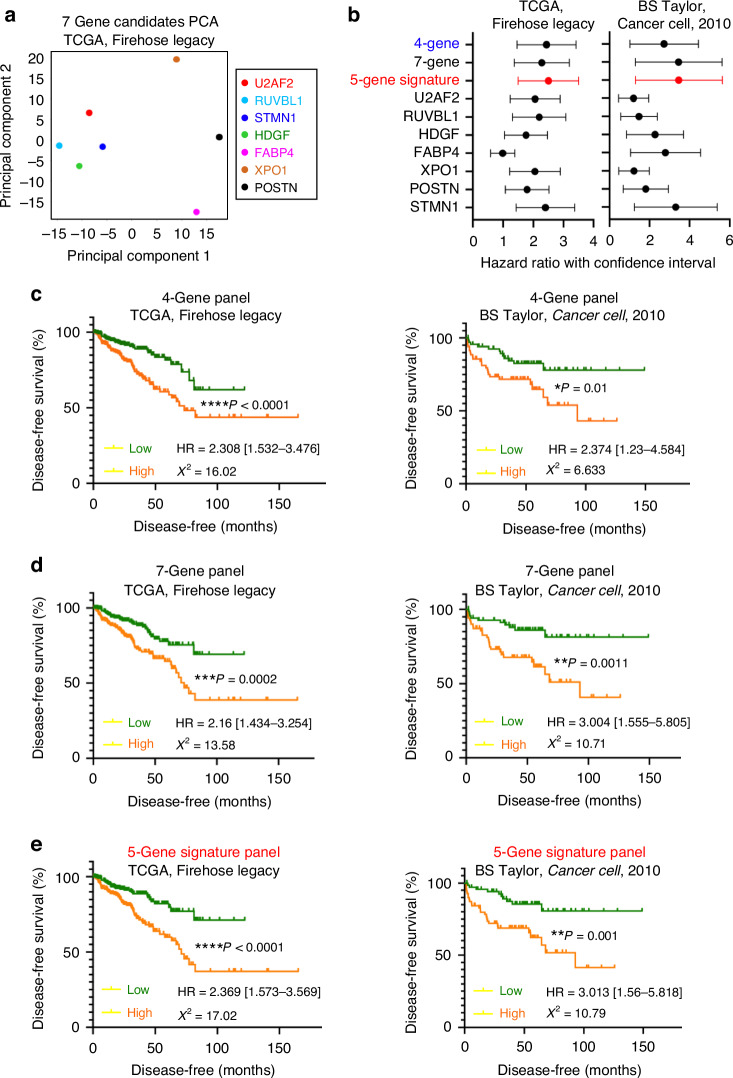
The 5-gene signature panel demonstrates improved ability to capture prostate cancer patient disease-free survival outcome. **a** Principal component analysis of the expression profiles of the seven candidates (U2AF2, RUVBL1, STMN1, HDGF, FABP4, XPO1, and POSTN) in the TCGA Firehose Legacy dataset. **b** Comparison of the hazard ratios of patient disease-free survival outcomes (with confidence intervals) using the seven individual candidates, the 4-gene panel (U2AF2, RUVBL1, STMN1, HDGF), the 7-gene panel, and the 5-gene signature panel (U2AF2, RUVBL1, STMN1, HDGF, and FABP4). The left panel shows the hazard ratios from the TCGA Firehose Legacy dataset, and the right panel compares the hazard ratios from the BS Taylor, *Cancer Cell*, 2010 dataset. **c** Kaplan–Meier plots that show the association between the expression levels of the 4-gene panel (U2AF2, RUVBL1, STMN1, and HDGF) and patient disease-free survival outcomes in the TCGA Firehose Legacy dataset (left) and the BS Taylor, *Cancer Cell*, 2010 dataset (right). For the right panel, *N* = 245 for the low-expression group and *N* = 246 for the high-expression group. For the left panel, *N* = 70 for both the high- and low-risk groups. The high and low-expression groups are determined assuming equal contributions of the four genes, and the median level was used as the cutoff threshold. The Log-rank *P*-value, hazard ratio (HR) with confidence intervals, and the chi-square statistics (${\chi }^{2}$) are labelled correspondingly. **d** Kaplan–Meier using the same datasets as (**c**) but with the 7-gene panel (U2AF2, RUVBL1, STMN1, HDGF, FABP4, XPO1, and POSTN). **e** Kaplan–Meier plots using the same datasets as (**c**) but with the 5-gene signature panel (U2AF2, RUVBL1, STMN1, HDGF, and FABP4). For all, *P < 0.05, **P < 0.01, ***P < 0.001, and ****P < 0.0001.

To develop a metastasis signature panel to best predict patient outcome, we further assessed whether different combinations of the seven candidates would achieve improved prediction of worse outcome relative to individual candidates. To determine the best combinations, we first performed principal component analysis on the expression profiles of the seven candidates in the TCGA Firehose Legacy dataset (Fig. 5a). We illustrated that four of the seven candidates (U2AF2, RUVBL1, STMN1, HDGF) have expression profiles in a cluster, suggesting that these candidates exhibit similar profiles that associate with similar features (Fig. 5a). To identify metastasis signature panel, we assessed the hazard ratios and statistical significance captured by a variety of combinations using Kaplan–Meier plots in the TCGA Firehose Legacy and the BS Taylor, *Cancer Cell*, 2010 datasets (Fig. 5b–e, Supplementary Fig. S4, S5). We identified that the 4-gene panel (U2AF2, RUVBL1, STMN1, HDGF) achieved an improved prediction in the TCGA Firehose Legacy dataset relative to individual candidates (Figs. 3, 5c-left). However, this 4-gene panel did not improve prediction in the BS Taylor, *Cancer Cell*, 2010 dataset since both STMN1 (*P* = 0.0016, HR = 2.879 [1.493–5.552]) and FABP4 (*P* = 0.0079, HR = 2.434 [1.263–4.69]) achieved better statistical significance and hazard ratios relative to the combined panel (*P* = 0.01, HR = 2.374 [1.23–4.584]) (Figs. 3f-right, g-right, 5b, c-right). To improve the separation between patient survival outcomes, we combined all 7 genes to capture more relative risk by including more features (Fig. 5b, d). However, the 7-gene panel only improved outcome prediction in the BS Taylor, *Cancer Cell*, 2010 dataset and is not consistent in the TCGA Firehose Legacy dataset (Fig. 5b, d). To prevent adding additional features that are subtractive for the prediction, we added features from the additional three candidates, FABP4, XPO1, and POSTN, to the 4-gene panel to find the optimal combination that best captures patient disease-free survival outcomes. After assessing all 5-gene and 6-gene panels, we discovered a combination that consistently improved disease-free survival outcome prediction in both TCGA Firehose Legacy and BS Taylor, *Cancer Cell*, 2010 (Fig. 5b–e, Supplementary Fig. S4, S5). The combination that demonstrated the best disease-free survival prediction was the 5-gene panel comprised of U2AF2, RUVBL1, STMN1, HDGF, and FABP4 (Fig. 5e). In both datasets, this combination achieved a separation with smaller *P*-values relative to all individual candidates, suggesting its ability to achieve a lower false positive rate in outcome prediction (Figs. 3a–c, f, g, 5e). The improvement of the statistical significance of this 5-gene signature panel relative to all single candidates in terms of *P*-values and ${\chi }^{2}$ statistics was also the most consistent across the two datasets when compared to all other combinations (Fig. 5c–e, Supplementary Fig. S4, S5). Additionally, this 5-gene panel also consistently captured more relative risks in its hazard ratios relative to all other combinations in both datasets, suggesting not only more confident, statistically significant predictions, but also increased risks association with an elevation in its expression (Fig. 5b–e, Supplementary Fig. S4, S5).

We then attempted to improve this 5-gene signature by using elastic net model to find weighted coefficients for the signature genes. We used the glmnet package in R to generate three elastic net models. Model 1 was trained in the TCGA Firehose Legacy dataset. Model 2 was trained in the BS Taylor, *Cancer Cell*, 2010 dataset, and model 3 was trained on the two datasets combined. However, we did not observe significant improvement in the weighted models relative to the original equally weighted 5-gene signature panel (Fig. 5e, Supplementary Fig. S6). Models 1 and 2 only significantly improved prediction in the datasets that they were trained in, which suggests overfitting to their training datasets (Supplementary Fig. S6A, B, Fig. 5e). Model 3 generated comparable results as the original 5-gene signature panel in both TCGA Firehose Legacy (model 3 HR = 2.52, unweighted HR = 2.37) and BS Taylor, *Cancer Cell*, 2010 datasets (model 3 HR = 3.28, unweighted HR = 3.01) (Supplementary Fig. S6C, Fig. 5e). However, the original unweighted signature demonstrates a better separation between high and lor groups in the Kaplan–Meier plots. Thus, the unweighted 5-gene signature panel is selected for further analyses.

### The 5-gene signature panel also correlates with metastatic prostate cancer in additional patient datasets

With the 5-gene prostate cancer metastasis signature panel comprised of U2AF2, RUVBL1, STMN1, HDGF, and FABP4, we further assessed the power of prediction of this gene signature panel in two different, independent public patient datasets (Grasso CS, *Nature*, 2012; Varambally S CS, *Cancer Cell*, 2005) [20, 21]. The Grasso CS, *Nature*, 2012 dataset included 28 benign prostate tissues, 59 localised prostate cancer tissues, and 35 metastatic castration-resistant prostate cancer (mCPRC) tissues [20]. In this dataset, 4 of the 5 signature genes (except FABP4) displayed a positive association with the onset of mCRPC relative to benign prostate and localised prostate cancer groups (U2AF2 *P* = 1.71 × 10^−9^, 8.75 × 10^−12^; RUVBL1 *P* = 1.13 × 10^−12^, 4.15 × 10^−9^; HDGF *P* = 2.06 × 10^−8^, 3.36 × 10^−11^; STMN1 *P* = 3.89 × 10^−7^, 2.49 × 10^−11^; FABP4 *P* = 0.0606, 0.1268) (Supplementary Fig. 7A–E). The Varambally S CS, *Cancer Cell*, 2005 dataset included six benign prostate, seven localised prostate cancer, and six metastatic prostate cancer samples [21]. The increased expression of the metastasis group relative to localised prostate cancer did not reach statistical significance in U2AF2 (*P* = 0.090), STMN1 (*P* = 0.087), and FABP4 (*P* = 0.093), potentially due to small sample sizes (Supplementary Fig. S7F–H). However, we still observed a statistically significant increase in the expressions of RUVBL1 (*P* = 0.0073, 0.012) and HDGF (*P* = 0.025, 0.025) when comparing the metastasis group to both benign and localised groups (Supplementary Fig. S7I, J). In addition, while the elevation of FABP4 in metastasis relative to benign prostate tissues did not reach statistical significance (*P* = 0.15), the expression of U2AF2 (*P* = 0.013) and STMN1 (*P* = 0.011) was significantly increased in the metastasis group relative to the benign group (Supplementary Fig. S7F–H).

**Fig. 6:**
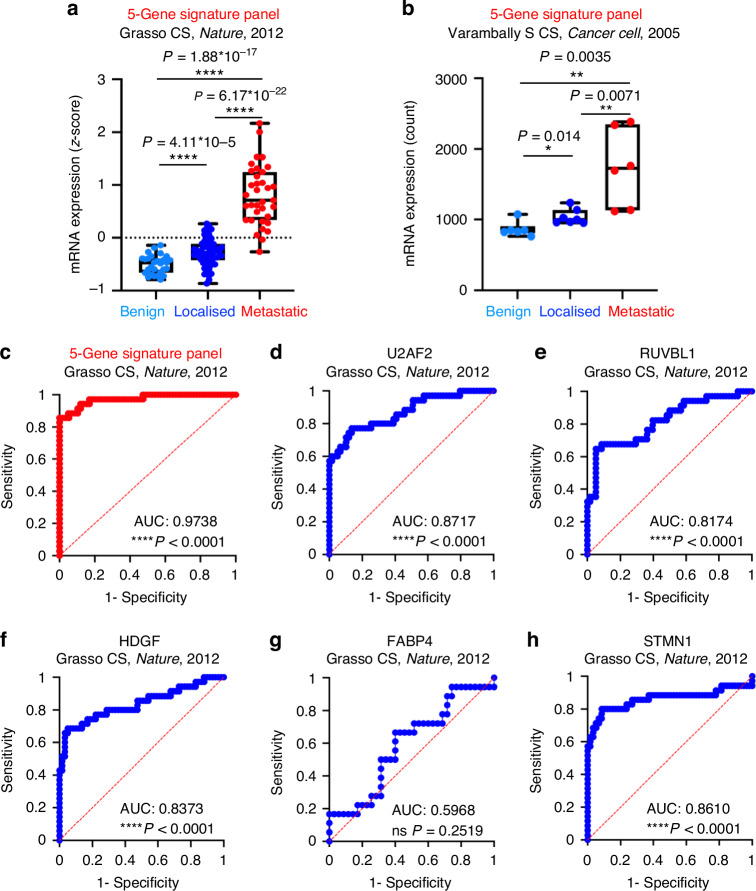
The 5-gene signature panel also displays improved ability to predict prostate cancer progression and the onset of prostate cancer metastasis in other independent datasets. **a** Box and whisker plot that shows the mRNA expression profile (z-score) of the 5-gene signature panel in the Grasso CS, *Nature*, 2012 dataset. This dataset is composed of 28 benign prostate samples, 59 localised prostate cancer samples, and 35 metastatic prostate cancer samples. The combined 5-gene expressions are calculated assuming equal contributions from the five signature genes. **b** Box and whisker plot that shows the mRNA expression profile of the 5-gene signature using the Varambally S CS, *Cancer Cell*, 2005 dataset. This dataset includes six benign prostate samples, seven localised prostate cancer tumours, and 6 metastatic prostate cancer samples. For all comparisons between the means of the two groups, Student’s *t*-test is performed with ns = non-significant, *P < 0.05, **P < 0.01, ***P < 0.001, and ****P < 0.0001. The corresponding *P*-values are labelled accordingly. **c** Receiver-operating characteristic (ROC) analysis of the 5-gene signature panel in the Grasso CS, *Nature*, 2012 dataset. In this analysis, *N* = 35 for patients with prostate cancer metastasis and *N* = 59 for patients with localised prostate cancer. The area under the curve (AUC) and the *P*-value are labelled. **d**–**h** Receiver-operating characteristic (ROC) analysis using each of the 5 single signature genes.

After characterising the expression profiles of the individual signature candidates in different stages of prostate cancer progression, we also tested the ability of the 5-gene signature panel to separate the metastatic group from the benign and localised groups (Fig. 6a, b). In both datasets, the 5-gene signature panel achieved improved separation between the metastasis and the localised groups relative to all individual candidates (Fig. 6a, b, Supplementary Fig. S7A–J). In the Grasso CS, *Nature*, 2012 dataset, the 5-gene signature panel displayed improved statistical significance when comparing the metastasis group to both benign (5-gene *P* = 1.88 × 10^−17^; single gene U2AF2 *P* = 1.71 × 10^−9^, RUVBL1 *P* = 1.13 × 10^−12^, HDGF *P* = 2.06 × 10^−8^, STMN1 *P* = 3.89 × 10^−7^, FABP4 *P* = 0.061) and localised groups (5-gene *P* = 6.17 × 10^−22^; single gene U2AF2 *P* = 8.75 × 10^−12^, RUVBL1 *P* = 4.15 × 10^−9^, HDGF *P* = 3.36 × 10^−11^, STMN1 *P* = 2.49 × 10^−11^, FABP4 *P* = 0.1268) (Fig. 6a, Supplementary Fig. S7A–E). Similarly, analysis in the Varambally S CS, *Cancer Cell*, 2005 dataset revealed the same improvement when comparing the mean of the metastasis group to that of the benign (5-gene *P* = 0.0035; single gene U2AF2 *P* = 0.0132, STMN1 *P* = 0.0111, FABP4 *P* = 0.1475, RUVBL1 *P* = 0.0073, and HDGF *P* = 0.0247) and localised groups (5-gene *P* = 0.0071; single gene U2AF2 *P* = 0.0899, STMN1 *P* = 0.0868, FABP4 *P* = 0.0932, RUVBL1 *P* = 0.0124, HDGF *P* = 0.0246) (Fig. 6b, Supplementary Fig. S7F–J). These results suggest that the 5-gene signature panel displays a more reliable association with prostate cancer metastasis than any single candidate by outperforming all single candidates across datasets.

In addition, we performed Receiver-Operating Characteristic (ROC) analysis in the Grasso CS, *Nature*, 2012 dataset (Fig. 6c–h). Our results demonstrated that the 5-gene signature panel displays an area under the curve (AUC) of 97.38% (*P* < 0.0001) relative to that of the single candidates (U2AF2 87.17%, *P* < 0.0001; RUVBL1 81.74%, *P* < 0.0001; HDGF 83.73%, *P* < 0.0001; FABP4 59.68%, *P* = 0.25; and STMN1 86.1%, *P* < 0.0001) (Fig. 6c–h). The 5-gene signature panel also associates with biochemical recurrence in the TCGA Firehose Legacy dataset (*P* = 0.0002) and an independent dataset, Gerhauser, *Cancer Cell*, 2018 (*P* = 0.019) (Supplementary Fig. S8A–C) [22]. The 5-gene signature demonstrates improved statistical significance relative to all single candidates in the TCGA dataset (Supplementary Fig. S8A, B). While the 5-gene signature panel is not the most statistically significant in the Gerhhauser, *Cancer Cell*, 2018 dataset, it demonstrates the smallest 95% confidence interval (Supplementary Fig. S8A, 8D–H). With its consistent association with worse patient outcomes and the metastatic phenotype across multiple datasets, the 5-gene signature can assist in making more reliable prognostic predictions by reducing the variation and enhancing the statistical significance of the risks associated with its expression.

## Discussion

Our proteomic profiling and transcript-level analysis revealed a 5-gene signature panel associated with a worse clinical course and the onset of metastasis. The components of this 5-gene signature panel include U2AF2, RUVBL1, HDGF, FABP4, and STMN1. U2 Small Nuclear RNA Auxiliary Factor 2 (U2AF2) is a heterodimer comprised of U2AF65 and U2AF35 that serves as an essential pre-mRNA splicing factor critical for spliceosome assembly to the pre-mRNA branch site [24, 25]. Previous studies demonstrated the role of U2AF2 in alternative splicing, which can foster a variety of diseases, including cancer [26, 27]. Our study reveals that U2AF2 is highly expressed in prostate cancer patients with biochemical recurrence and worse disease-free survival. We also demonstrate that U2AF2 mRNA and protein are both elevated in prostate cancer metastasis relative to localised and benign prostate tissues. However, while U2AF2 has been implicated in some solid tumours, such as non-small cell lung cancer and glioma, its associations with prostate cancer and metastasis have yet to be elucidated [28, 29]. Further assessment of the role of U2AF2 in advanced, metastatic prostate cancers is needed to test its functional role in prostate cancer metastasis. RuvB like AAA ATPase 1 (RUVBL1) is an ATPase that can associate with various complexes and participate in various cellular processes, including chromatin remodelling and transcriptional regulation [30]. RUVBL1 has been reported to promote the invasion of breast and pancreatic cancers and increased proliferation and resistance in various solid tumours, including lung cancer and colorectal cancers [31–36]. In prostate cancer, RUVBL1 has been associated with Enzalutamide resistance [37]. A genome-wide association study also suggests that elevated RUVBL1 increases cell proliferation and tumour growth, further suggesting an association between RUVBL1 expression and prostate cancer progression [38]. HDGF, also known as hepatoma-derived growth factor, is a growth factor with both mitogenic and DNA-binding activity. HDGF has been implicated in angiogenesis, tumorigenesis, and worse disease prognosis in oral, bladder, and lung cancers [39–43]. While the prognostic value of HDGF has not yet been evaluated before, previous studies have shown that downregulation of HDGF inhibited migration and invasion of prostate cancer cells, and HDGF activated the MAPK/ERK pathway via KRAS and RhoA mediation in cell line models of prostate cancer [44–46]. Fatty acid binding protein 4 (FABP4) is an intracellular lipid-binding protein that regulates lipid trafficking and metabolism [47]. FABP4 is a biomarker for metabolic diseases and has been shown to promote proliferation, resistance, and metastasis in ovarian cancer, breast cancer, and prostate cancer [47, 48]. FABP4 can exert endocrine and exocrine effects as it is secreted from adipocytes and macrophages, and its association with prostate cancer supports further assessment of the regulatory relationships between these cells and prostate cancer progression and metastasis [49–51]. Lastly, stathmin (STMN1) is the best-performing single candidate in our signature panel with its highly significant association with biochemical recurrence, patient disease-free survival, and prostate cancer progression. While the function and targeting of STMN1 have been characterised in a variety of cancers ranging from lung and breast cancers to leukaemia, the functional role of STMN1 in prostate cancer metastasis remains unknown [52–55]. Our results warrant further assessment of the mechanism of action behind these candidates. In addition, this study only included candidates that associate with prostate cancer metastasis on both protein and mRNA levels to enable a wider application across various clinical platforms that utilise protein or RNA detection. However, there could be genes that were not chosen in this study that are only deregulated at the protein level, not at the RNA level and vice versa. As a result, further assessment of other candidates from our proteomic analysis, as well as the other RNA candidates implied in the transcript-level analyses, is needed for additional biomarker identification.

Our study reports a new 5-gene metastasis signature panel that correlates with an increased risk of worse patient disease-free survival. This suggests that this combined 5-gene signature panel can be used in prognosis prediction to identify patients who are likely to experience poor survival outcomes. This 5-gene metastasis signature panel also correlates with prostate cancer metastasis relative to localised prostate cancer and benign prostate tissues. Compared to the expression profiles of individual candidates, the combined 5-gene panel separates the metastasis prostate cancer group from localised, primary prostate cancers with improved statistical significance. This suggests that an increase in the expression of the 5-gene panel represents a higher risk of metastasis with a reduced probability of false positives relative to an increase in any single candidate. Further assessment of this 5-gene panel as predictors and effectors of prostate cancer metastasis in additional large, independent prostate cancer patient cohorts is warranted.

## Supplementary information

Supplementary Figures S1-8 with Figure Legends

## Data Availability

All patient datasets used in this study can be accessed via cBioPortal (https://www.cbioportal.org/) or on Gene Expression Omnibus (GDS2545/ GSE6919, GSE35988, and GSE3325). Proteomic data have been deposited at PRIDE and are publicly available with the identifier PXD056300. Any additional information required to reanalyze the data reported in this paper is available from the corresponding author upon request.

## Code availability

All Python and R codes utilised in this study can be accessed through the following three GitHub repositories: https://github.com/shen-michelle/5-gene-Metastasis.git, https://github.com/shen-michelle/5-gene-Metatasis-PCA.git, https://github.com/shen-michelle/5-gene-Metastasis-Weights.
