# Supplementary material for: Identification of a 5-gene signature panel for the prediction of prostate cancer progression
Source: Br J Cancer. 2024 Oct 14;131(11):1748–61. doi: 10.1038/s41416-024-02854-w (PMC11589118; doi:10.1038/s41416-024-02854-w)
Supplement: Supplementary file 1 — Supplementary Figures S1-8 with Figure Legends [file 41416_2024_2854_MOESM1_ESM.pdf]

Supplementary Figure S1

A

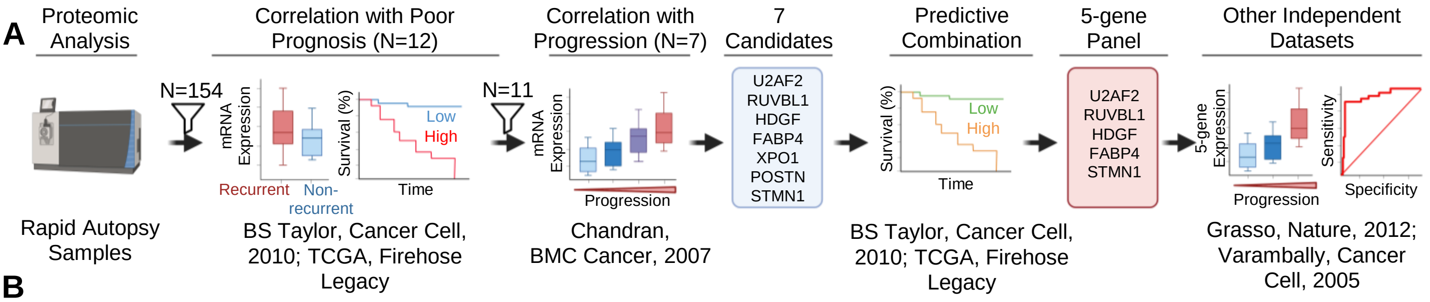

B

	Biochemical Recurrence				Worse Disease-Free Survival				Metastasis	
	TCGA Firehose Legacy		BS Taylor, Cancer Cell, 2010		TCGA Firehose Legacy		BS Taylor, Cancer Cell, 2010		Chandran UR, BMC Cancer, 2007	
Genes	Correlation	Statistical Significance	Correlation	Statistical Significance	Correlation	Statistical Significance	Correlation	Statistical Significance	Correlation	Statistical Significance
**U2AF2**	+	**P= 0.0045**	ns	P= 0.9483	+	**P= 0.0015**	ns	P= 0.8854	+	P= 0.0106
**RUVBL1**	+	**P= 0.0038**	+	P= 0.013	+	**P= 0.0005**	ns	P= 0.4560	+	P= 0.0508*
**HDGF**	+	P= 0.0424	ns	P= 0.1086	+	P= 0.0157	+	P= 0.0406	+	P< 0.0001
**FABP4**	ns	P= 0.3845	+	**P= 0.0005**	ns	P= 0.7441	+	**P= 0.0079**	+	P= 0.0005
**XPO1**	+	**P= 0.0021**	ns	P= 0.9424	+	**P= 0.0025**	ns	P= 0.8394	+	P< 0.0001
**POSTN**	+	P= 0.0443	+	**P= 0.0073**	+	P= 0.0116	ns	P= 0.1731	+	P= 0.0015
**STMN1**	+	**P= 0.0008**	+	**P< 0.0001**	+	**P= 0.0001**	+	**P= 0.0016**	+	P= 0.0316
**ASPN**	+	**P= 0.00022**	+	**P< 0.0001**	+	**P= 0.0008**	+	**P= 0.0050**	na	Non-available
**DDX39B**	+	**P= 0.0027**	ns	P= 0.0690	+	**P= 0.00051**	ns	P= 0.5036	ns	P= 0.1700
**CA2**	ns	P= 0.0912	+	P< 0.0001	+	P= 0.0042	+	P= 0.00067	ns	P= 0.2898
**H2AC1**	ns	P= 0.4953	+	**P= 0.0032**	+	**P= 0.00014**	+	**P= 0.0092**	na	Non-available
MACROH2A1	+	P= 0.0335	ns	P= 0.7452	+	P= 0.0097	ns	P= 0.9583	na	NA
CCT3	+	P= 0.0175	ns	P= 0.1412	ns	P= 0.9467	ns	P= 0.1389	na	NA
KPNB1	+	P= 0.0069	ns	P= 0.7860	ns	P= 0.6136	ns	P= 0.1962	na	NA
TKT	+	P= 0.0052	+	P= 0.0199	ns	P= 0.4012	ns	P= 0.3542	na	NA
GCN1	+	P= 0.0022	ns	P= 0.2716	ns	P= 0.3391	ns	P= 0.1789	na	NA
GART	+	P= 0.0066	ns	P= 0.9925	ns	P= 0.0562	ns	P= 0.2887	na	NA
LRPPRC	+	P= 0.0226	ns	P= 0.6774	ns	P= 0.5921	ns	P= 0.8979	na	NA
AIFM1	+	P= 0.0304	ns	P= 0.6003	ns	P= 0.0682	ns	P= 0.7426	na	NA
PABPC1	+	P= 0.00034	ns	P= 0.2979	ns	P= 0.8452	ns	P= 0.9863	na	NA
SLC25A5	+	P= 0.0458	ns	P= 0.1522	ns	P= 0.4257	ns	P= 0.8963	na	NA
SPTBN2	+	P= 0.0299	ns	P= 0.8035	ns	P= 0.3073	ns	P= 0.2055	na	NA
SNRNP200	+	P= 0.0192	ns	P= 0.1874	ns	P= 0.9300	ns	P= 0.2367	na	NA
YWHAZ	+	P= 0.0114	ns	P= 0.8341	ns	P= 0.3798	ns	P= 0.3091	na	NA
EPPK1	+	P= 0.0392	ns	P= 0.1296	ns	P= 0.7643	ns	P= 0.3164	na	NA
MTTP	ns	P= 0.7484	+	P= 0.0404	ns	P= 0.5879	ns	P= 0.0817	na	NA
PSAT1	ns	P= 0.4013	+	P= 0.0215	ns	P= 0.4247	ns	P= 0.1578	na	NA
HBA1	ns	P= 0.9218	+	P= 0.0447	ns	P= 0.6991	ns	P= 0.7046	na	NA
PCYOX1	-	P= 0.0050	ns	P= 0.1184	+	P< 0.0001	+	P= 0.0119	na	NA
DDX17	ns	P= 0.1594	-	P= 0.0068	+	P= 0.0253	ns	P= 0.3193	na	NA
TRA2B	ns	P= 0.5960	-	P= 0.0088	+	P= 0.0016	ns	P= 0.4454	na	NA
SRSF7	ns	P= 0.8974	-	P= 0.0013	+	P= 0.0209	+	P= 0.0011	na	NA
HNRNPA3	ns	P= 0.8541	-	P= 0.0310	+	P= 0.0089	+	P= 0.0149	na	NA
CMBL	ns	P= 0.0850	ns	P= 0.1780	+	P= 0.0396	ns	P= 0.9161	na	NA
SRSF9	ns	P= 0.2740	ns	P= 0.7244	+	P= 0.0064	ns	P= 0.5543	na	NA
NANS	-	P= 0.0016	-	P= 0.0122	+	P= 0.0411	+	P= 0.0180	na	NA
ACSL3	ns	P= 0.2082	ns	P= 0.1192	+	P= 0.0070	ns	P= 0.1394	na	NA
ARCN1	-	P= 0.0102	ns	P= 0.1294	+	P= 0.00076	ns	P= 0.2200	na	NA
CPNE1	ns	P= 0.2228	ns	P= 0.5936	+	P= 0.0065	ns	P= 0.4237	na	NA
H2AC20	ns	P= 0.8699	ns	P= 0.9049	+	P= 0.0188	ns	P= 0.2265	na	NA
ILF2	ns	P= 0.8762	ns	P= 0.3438	+	P= 0.0330	ns	P= 0.3376	na	NA
AOX1	-	P= 0.0171	-	P= 0.0036	+	P= 0.0147	+	P= 0.0071	na	NA
ALDH6A1	ns	P= 0.1385	ns	P= 0.4344	+	P= 0.0492	ns	P= 0.4330	na	NA
PDXDC1	ns	P= 0.7652	-	P= 0.0036	+	P= 0.0492	ns	P= 0.3782	na	NA
SCARB2	-	P= 0.0282	ns	P= 0.2355	+	P< 0.0001	+	P= 0.0306	na	NA
PAICS	ns	P= 0.1758	ns	P= 0.8023	+	P= 0.0459	ns	P= 0.5188	na	NA
HIBADH	ns	P= 0.3522	ns	P= 0.7059	+	P= 0.0195	ns	P= 0.5779	na	NA
TARDBP	ns	P= 0.8523	-	P= 0.0140	ns	P= 0.7943	+	P= 0.0226	na	NA
SUCLG2	ns	P= 0.9219	ns	P= 0.1429	ns	P= 0.5843	+	P= 0.0391	na	NA
HK1	-	P= 0.0339	-	P= 0.00075	ns	P= 0.2972	+	P= 0.00031	na	NA
NAXE	ns	P= 0.8062	ns	P= 0.0785	ns	P= 0.8098	+	P= 0.0194	na	NA
ASS1	ns	P= 0.7675	ns	P= 0.2800	ns	P= 0.6995	+	P= 0.0426	na	NA
RDX	ns	P= 0.8216	ns	P= 0.2547	ns	P= 0.2755	+	P= 0.0209	na	NA

Supplementary Figure S1. Screening of Proteomic Candidates in Prostate Cancer Patient Datasets Reveals 7 Candidates that Correlates with Biochemical Recurrence, Worse Disease-free Survival, and Prostate Cancer Metastasis

(A) Workflow schematic of the selection untl the final 5-gene signature panel. Created with BioRender.com (https://biorender.com). A filter of FDR< 0.05 annd |fold change|>1.5 was applied to the proteomic candidates discovered from patient rapid autopsy samples, revealing 154 proteins increased in prostate cancer metastasis. Then, TCGA Firehose Legacy dataset and BS Taylor, Cancer Cell, 2010 dataset were used to assess their correlation with biochemical recurrence and worse disease-free survival. This revealed 12 candidates with positive correlation in with both biochemical recurrence and worse disease-free survival. To reduce dataset-specific candidates, only 11 candidates were selected since they consistently associate with biochemical recurrence and/or worse disease-free survival in both datasets or because they satisfied a more stringent FDR of P< 0.01 in their association with biochemical recurrence and worse disease-free survival. These 11 candidates are selected for analysis in the Chandran UR, BMC Cancer, 2007 dataset to confirm an elevation in prostate cancer metastasis, revealing the final 7 candidates. Then, combinations of the 7 candidates were tested in the TCGA Firehose Legacy and BS Taylor, Cancer Cell, 2010 datasets to find the combination that most consistently predicts worse survival. The derived 5-gene panel was assessed in two other independent datasets (Grasso, Nature, 2012; Varambally, Cancer Cell, 2005). (B) Summary table shows the compiled data from the 53 proteomic candidates that exhibits positive associations with biochemical recurrence and/or worse disease-free survival in at least one of the two datasets used. Statistically significant positive correlations with biochemical recurrence are highlighted in red. Statistically significant positive correlations with worse disease-free survival are highlighted in yellow. Tests that satisfied the FDR of P<0.01 are bolded. Out of the 11 genes analyzed in the Chandran UR, BMC Cancer, 2007 dataset, the 7 candidates (U2AF2, RUVBL1, HDGF, FABP4, XPO1, POSTN, and STMN1) that confirmed an association with prostate cancer progression and metastasis are selected

as the final 7-gene candidates. Genes that are unavailable in the Chandran UR, BMC Cancer, 2007 dataset are labeled accordingly.

Supplementary Figure S2

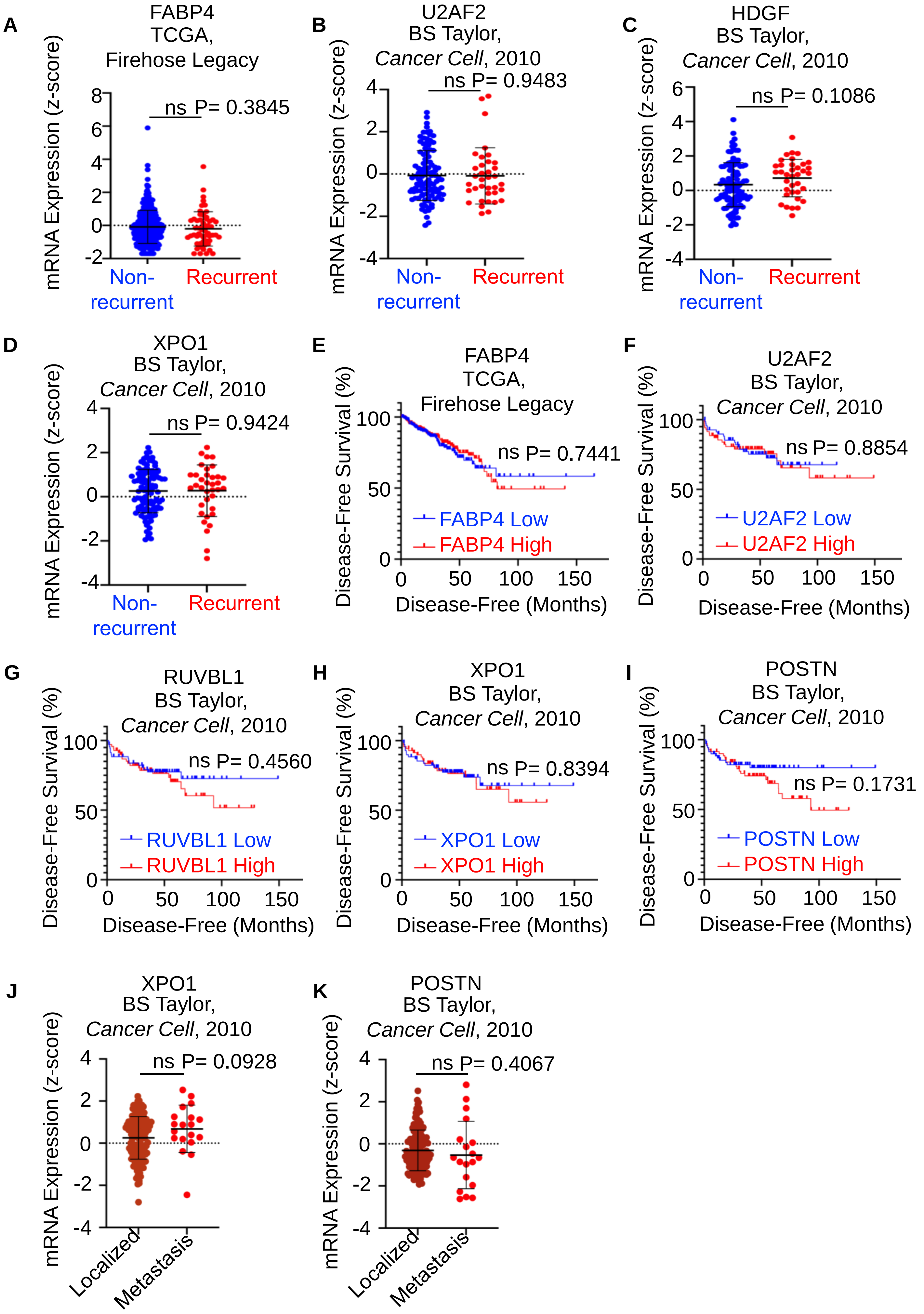

Supplementary Figure S2. Non-significant Associations Highlight Variability Between Datasets when Assessing the Correlation between the 7 Candidates, Biochemical Recurrence, Patient Disease-free Survival, and Prostate Cancer Metastasis

(A) Expression levels of FABP4 in recurrent (N= 58) vs non-recurrent (N= 371) prostate cancer in the TCGA Firehose Legacy dataset. (B) Expression levels of U2AF2 in recurrent (N= 36) vs non-recurrent (N= 104) prostate cancer in the BS Taylor, Cancer Cell, 2010 dataset. (C-D) Expression levels of HDGF (C) and XPO1 (D) in the BS Taylor, Cancer Cell, 2010 dataset described in (B). (E) Kaplan Meier plot shows the association between FABP4 expression levels and prostate cancer disease-free survival outcomes in the TCGA Firehose Legacy dataset. The FABP4 high (N= 246) and low groups (N= 245) were formed using the median FABP4 expression level as the cutoff threshold. The Log-rank P-value is labeled on the graph. (F-I) U2AF2 (F), RUVBL1 (G), XPO1 (H), POSTN (I) expression levels and patient disease-free survival outcomes in the BS Taylor, Cancer Cell, 2010 dataset. The high (N= 70) and low (N= 70) groups were formed using the median expression level as the cutoff. The Log-rank P-values are labeled accordingly. (J-K) Expression levels of XPO1 (J), and POSTN (K) in loccalized prostate cancer (N= 131) and prostate cancer metastasis (N= 19) using the BS Taylor, Cancer Cell, 2010 dataset. Student’s t-tests are performed, and the P-values are labeled on the plots.

Supplementary Figure S3

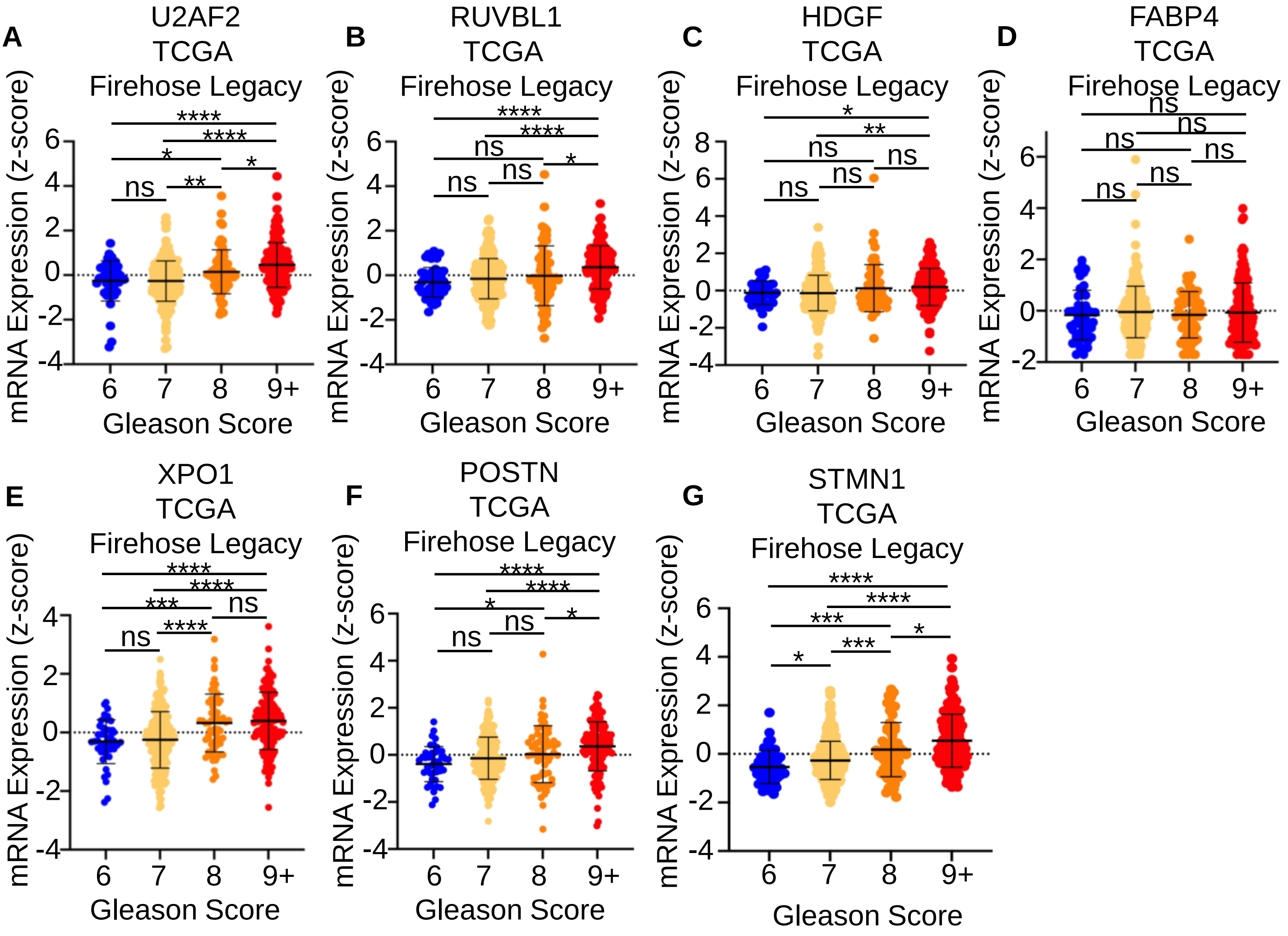

Supplementary Figure S3. The expression of 6 of the 7 Candidates are Associated with Higher Gleason Score in Prostate Cancer Patients

(A) U2AF2 mRNA expression profile across various Gleason scores at radical prostatectomy. U2AF2 expression and patient Gleason scores are obtained from the TCGA Firehose Legacy dataset, and N= 45 for Gleason score of 6, N= 246 for Gleason score of 7, N= 64 for Gleason score of 8, N= 141 for Gleason score of 9+. (B-G) The same analysis performed in (A) but using RUVBL1 (B), HDGF (C), FABP4 (D), XPO1 (E), POSTN (F), and STMN1 (G). For all comparisons between 2 groups, the Student's t-test was performed with ns= non-significant, *P< 0.05, **P< 0.01, ***P< 0.001, and ****P< 0.0001.

Supplementary Figure S4

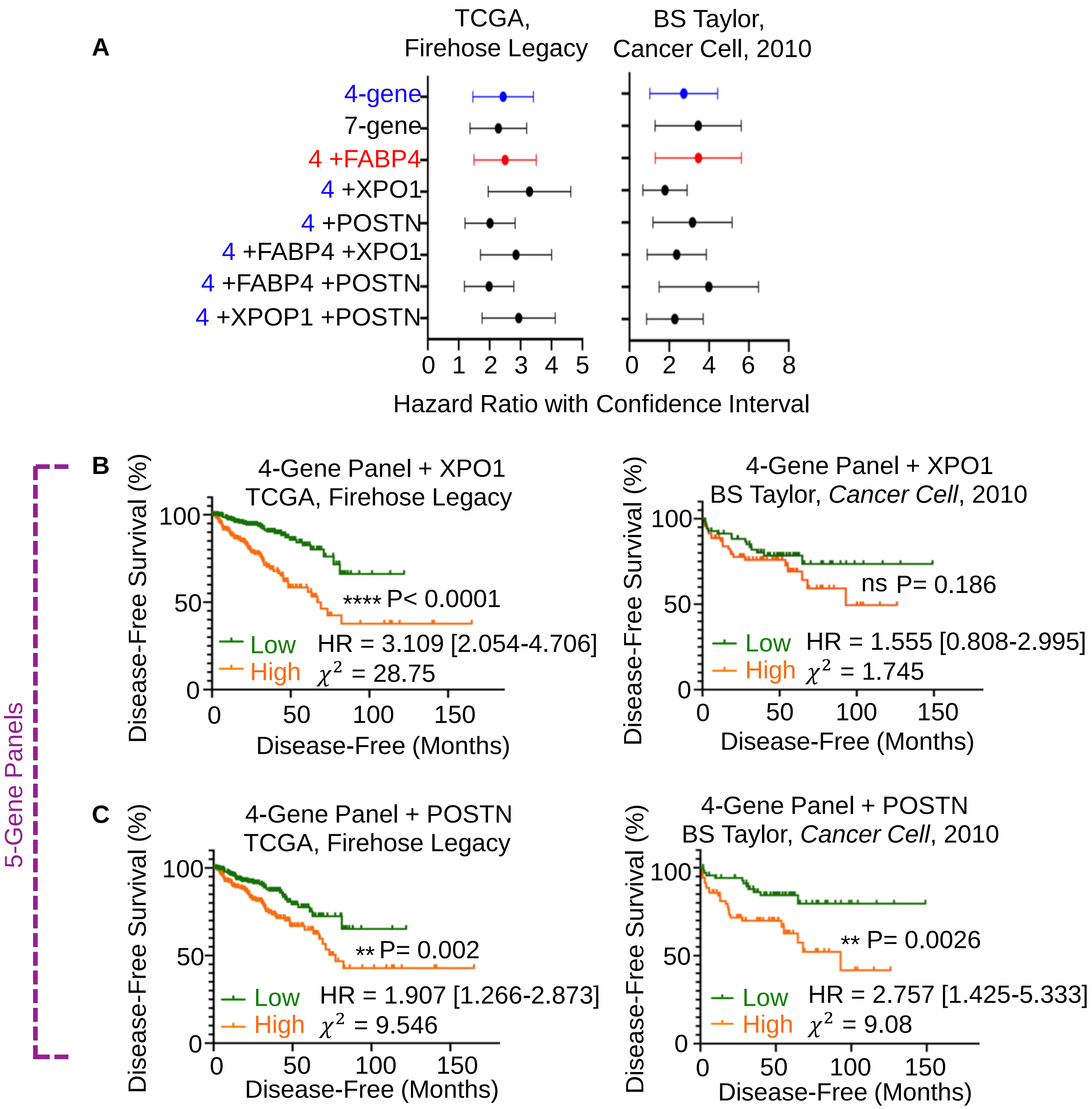

Supplementary Figure S4. Kaplan Meier Plots of Prostate Cancer Patient Disease-free Survival Outcome using the Additional 5-gene Combinations

(A) Kaplan Meier plots using the 5-gene panel derived by adding XPO1 to the 4-gene panel from Figure 5 (U2AF2, RUVBL1, STMN1, and HDGF). In the TCGA Firehose Legacy dataset (left), N= 246 for the high-risk group and N= 245 for the low-risk group. In the BS Taylor, Cancer Cell, 2010 dataset (right), N= 70 for both high and low-risk groups. The high and low expression groups were derived assuming equal contributions from the individual expression levels of the five genes and using the median level as the cutoff threshold. The Log-rank P-values, hazard ratios (HR) with confidence intervals, and the chi-square statistics (χ^2) are labeled accordingly. (B) The same analysis performed in (A) but using the 5-gene signature that includes POSTN (U2AF2, RUVBL1, STMN1, HDGF, and POSTN). For all Log-rank P-values, ns= non-significant, *P< 0.05, **P< 0.01, ***P< 0.001, and ****P< 0.0001.

Supplementary Figure S5

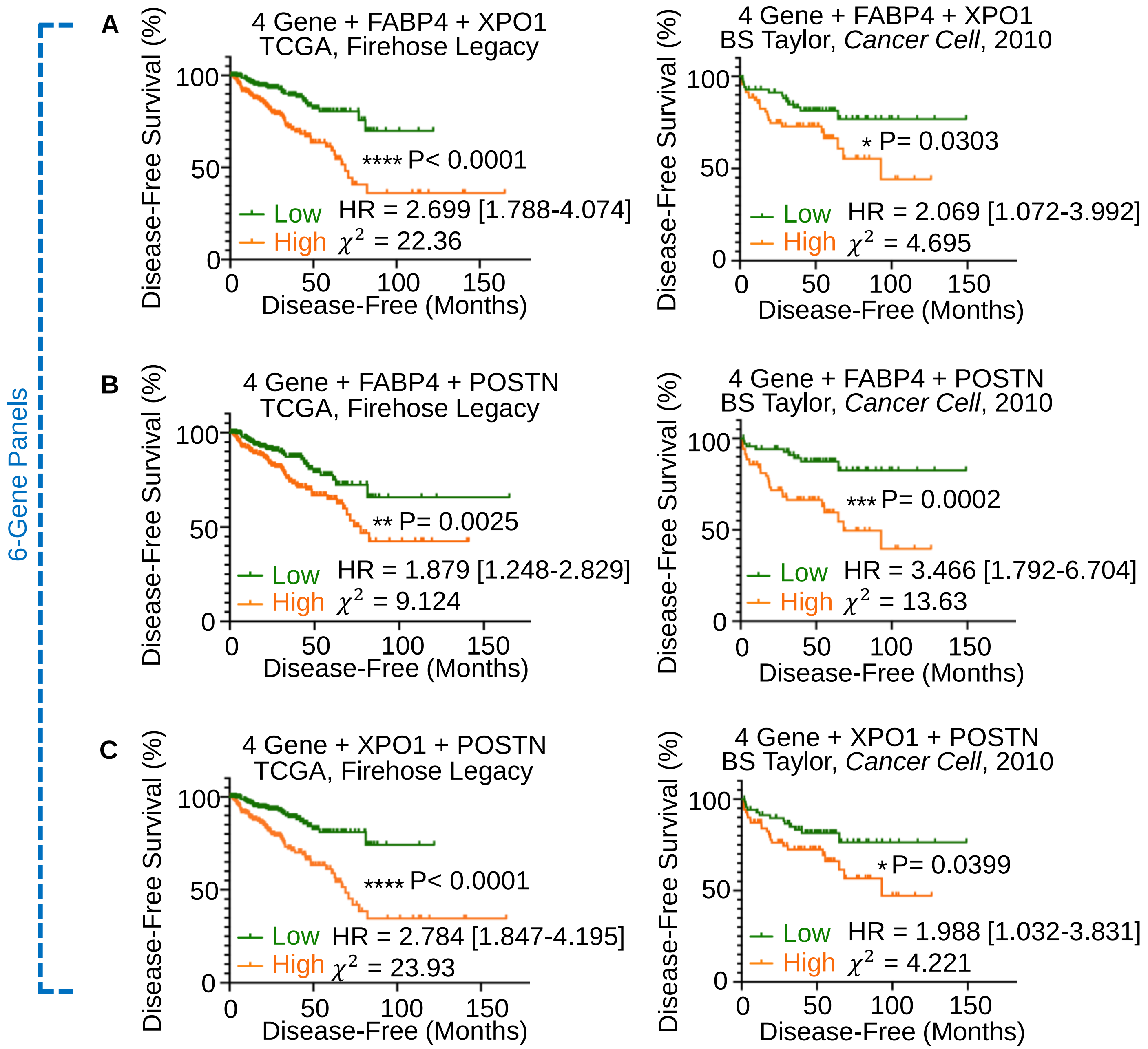

Supplementary Figure S5. Kaplan Meier Plots of Prostate Cancer Patient Disease-free Survival Outcome using all 6-gene Combinations

(A) Kaplan Meier plots using the 6-gene panel derived by adding FABP4 and XPOP1 to the 4-gene panel described in Figure 5 (U2AF2, RUVBL1, STMN1, and HDGF). In the TCGA Firehose Legacy dataset (left), N= 246 for the high-risk group and N= 245 for the low-risk group. In the BS Taylor, Cancer Cell, 2010 dataset (right), N= 70 for both high and low-risk groups. The high- and low-risk groups were derived by averaging the individual expression levels of the six genes and using the median level as the cutoff threshold. The Log-rank P-values, hazard ratios (HR) with confidence intervals, and the chi-square statistics (χ^2) are labeled accordingly. (B) The same analysis performed in (A) but using the 6-gene panel of U2AF2, RUVBL1, STMN1, HDGF, FABP4, and POSTN. (C) The same analysis performed in (A) but using the 6-gene panel of U2AF2, RUVBL1, STMN1, HDGF, XPO1, and POSTN. For all Log-rank P-values, ns= non-significant, *P< 0.05, **P< 0.01, ***P< 0.001, and ****P< 0.0001.

Supplementary Figure S6

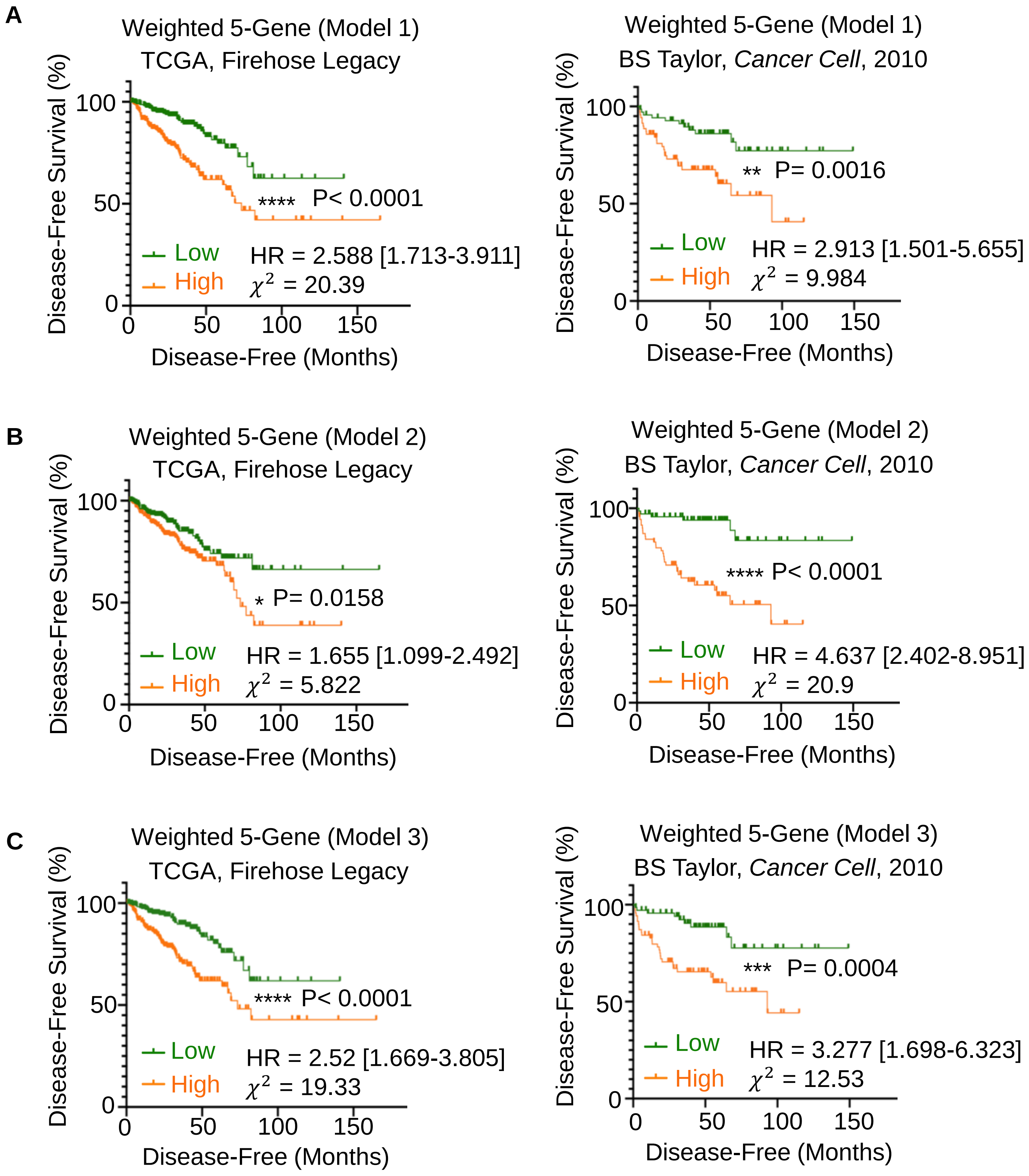

Supplementary Figure S6. Kaplan Meier Plots of Prostate Cancer Patient Disease-free Survival Outcome using Weighted 5-gene Combinations

(A) Elastic net model fitting was performed using the 5 signature genes (U2AF2, RUVBL1, HDGF, FABP4, and STMN1) as variables. Three models were generateds with different training datasets. Model 1 was trained on the TCGA, Firehose Legacy dataset (with 400 non-recurrent and 91 recurrent prostate cancer samples) to best predict biochemical recurrence outcome. The coefficients obtained from model 1 were 0.21995 for U2AF2, 0.11618 for RUVBL1, -0.06077 for HDGF, 0 for FABP4, and 0.31602 for STMN1. Model 1 was tested in both the TCGA, Firehose Legacy dataset and the BS Taylor, Cancer Cell, 2010 dataset. The median score was used as the cut off for the low and high groups. (B) Model 2 was trained in the BS Taylor, Cancer Cell, 2010 dataset with 104 non-recurrent and 36 recurrent prostate cancer samples. The coefficients for model 2 were -0.41372 for U2AF2, 0.15510 for RUVBL1, 0.20957 for HDGF, 0.31032 for FABP4, and 0.45953 for STMN1. (C) Model 3 was trained on both TCGA, Firehose Legacy and BS Taylor, Cancer Cell, 2010 datasets combined. The coefficients were 0.04864 for U2AF2, 0.09297 for RUVBL1, -0.02813 for HDGF, 0.06348 for FABP4, and 0.41772 for STMN1. For all Log-rank P-values, ns= non-significant, *P< 0.05, **P< 0.01, ***P< 0.001, and ****P< 0.0001.

Supplementary Figure S7

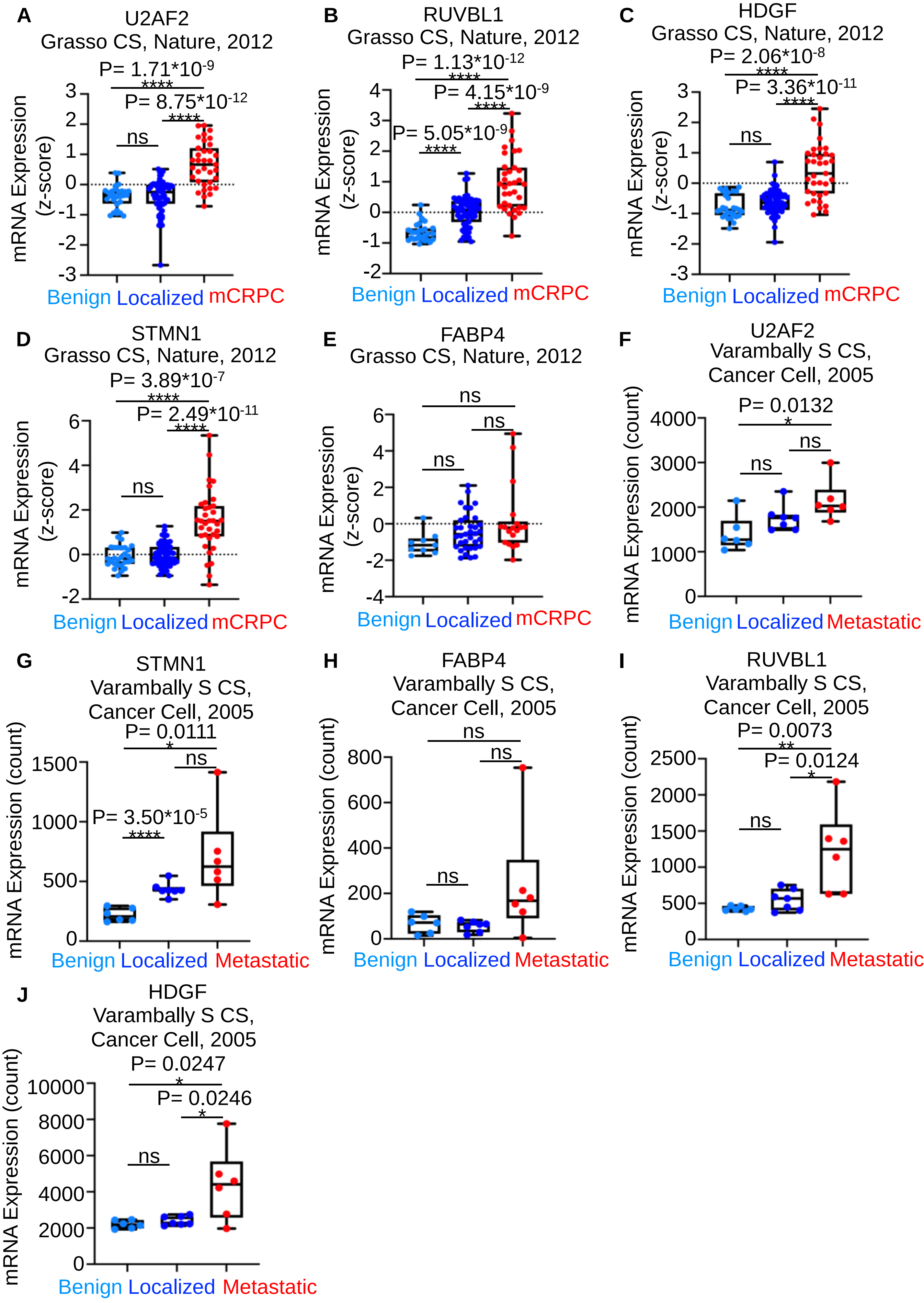

Supplementary Figure S7. Assessing the Association between the Expression of the Single Signature Genes and Prostate Cancer Progression

(A-E) Box and whisker plots display the mRNA expression profiles (z-score) of all 5 individual signature genes from the 5-gene signature panel (U2AF2, RUVBL1, HDGF, STMN1, and FABP4) in the Grasso CS, Nature, 2012 dataset. This dataset includes 28 samples of benign prostate tissues, 59 samples of localized prostate cancer, and 35 samples of metastatic castration resistant prostate cancer (mCRPC). (F-J) Box and whisker plots display the mRNA expression levels (in counts) of the same five individual signature genes in the Varambally S CS, Cancer Cell, 2005 dataset. This dataset includes six samples of benign prostate tissues, seven samples of localized prostate tumors, and six samples of metastatic prostate cancer tumors. For all comparisons between two groups, Student's t-tests were performed with ns= non-significant, *P< 0.05, **P< 0.01, ***P< 0.001, and ****P< 0.0001. The corresponding P-values were computed and labeled correspondingly.

Supplementary Figure S8

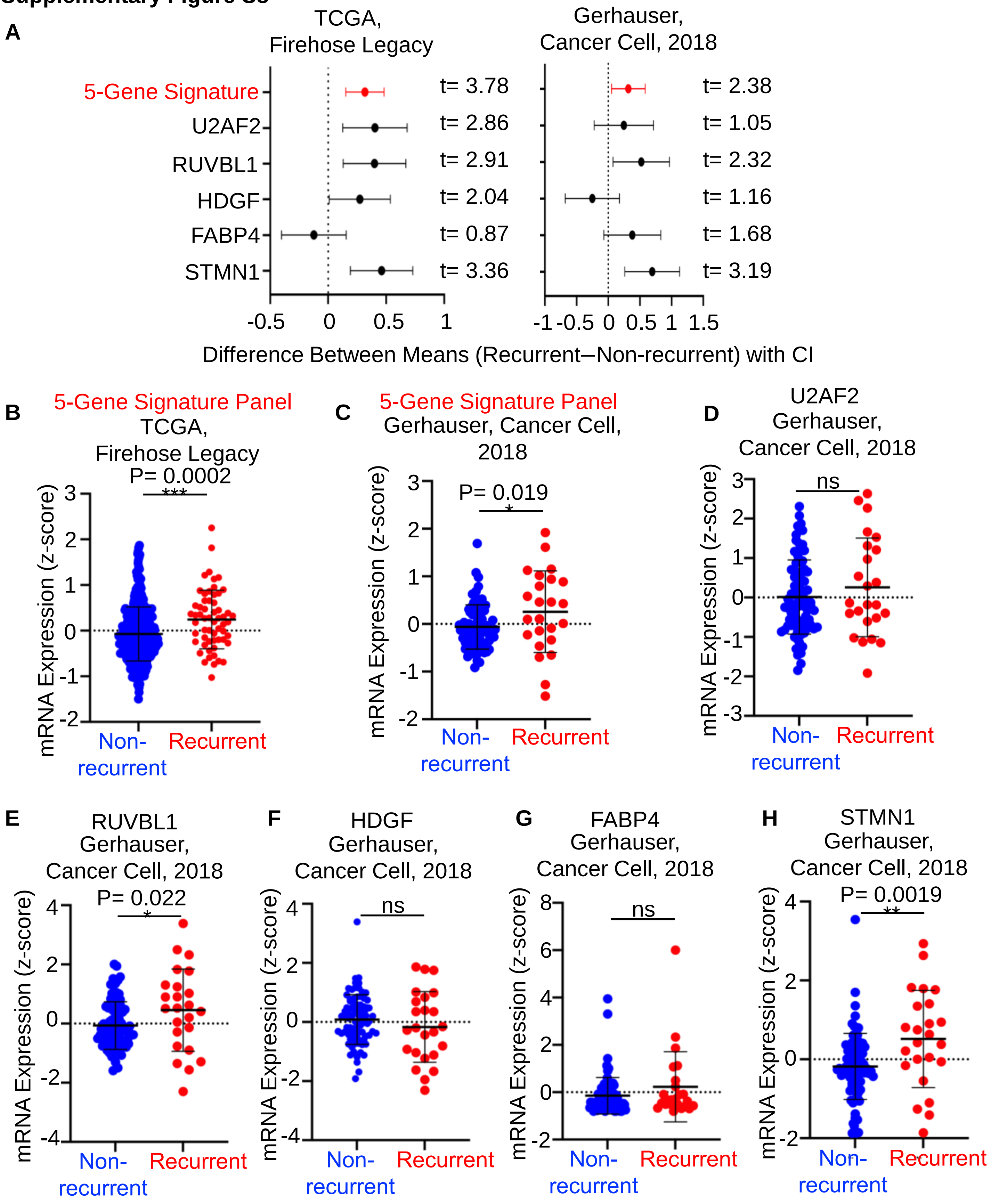

Supplementary Figure S8. The 5-gene Signature Panel also Separates Recurrent Prostate Cancer from Non-recurrent Prostate Cancer

(A) Comparison of the difference between means (recurrent – non-recurrent) with 95% confidence intervals. The t statistics are reported next to each row, and the 5-gene signature panel is compared too all 5 of the inndividual candidates U2AF2, RUVBL1, HDGF, FABP4, STMN1. The left panel shows the analyses from the TCGA Firehose Legacy dataset (with 400 non-recurrent and 91 recurrent prostate cancer samples), and the right panel shows the analyses from another independent dataset Gerhauser, Cancer Cell, 2018 (with 81 non-recurrent and 24 recurrent samples). (B-C) Dot plots that show the separation between recurrent and non-recurrent groups using the 5-gene signature panel in (B) the TCGA Firehose Legacy dataset and te (C) Gerhauser, Cancer Cell, 2018 dataset. (D-H) Dot plots showing the association between individual candidates (D) U2AF2, (E) RUVBL1, (F) HDGF, (G) FABP4, (H) STMN1 and biochemical recurrence. For all comparisons between two groups, Student's t-tests were performed with ns= non-significant, *P< 0.05, **P< 0.01, and ***P< 0.001. The corresponding P-values were computed and labeled correspondingly.
